# Old plasma dilution reduces human biological age: a clinical study

**DOI:** 10.1007/s11357-022-00645-w

**Published:** 2022-08-24

**Authors:** Daehwan Kim, Dobri D. Kiprov, Connor Luellen, Michael Lieb, Chao Liu, Etsuko Watanabe, Xiaoyue Mei, Kaitlin Cassaleto, Joel Kramer, Michael J. Conboy, Irina M. Conboy

**Affiliations:** 1 Department of Bioengineering and QB3 Institute, University of California, Berkeley, CA 94720 USA; 2 Global Apheresis, Mill Valley, CA USA; 3 Biophysics, University of California, Berkeley, Berkeley, CA 94720 USA; 4 Brain Aging Center, UCSF, San Francisco, USA

**Keywords:** Aging, Rejuvenation, Plasmapheresis, Proteomics, Lymphoid/myeloid markers, Biological noise

## Abstract

This work extrapolates to humans the previous animal studies on blood heterochronicity and establishes a novel direct measurement of biological age. Our results support the hypothesis that, similar to mice, human aging is driven by age-imposed systemic molecular excess, the attenuation of which reverses biological age, defined in our work as a deregulation (noise) of 10 novel protein biomarkers. The results on biological age are strongly supported by the data, which demonstrates that rounds of therapeutic plasma exchange (TPE) promote a global shift to a younger systemic proteome, including youthfully restored pro-regenerative, anticancer, and apoptotic regulators and a youthful profile of myeloid/lymphoid markers in circulating cells, which have reduced cellular senescence and lower DNA damage. Mechanistically, the circulatory regulators of the JAK-STAT, MAPK, TGF-beta, NF-κB, and Toll-like receptor signaling pathways become more youthfully balanced through normalization of TLR4, which we define as a nodal point of this molecular rejuvenation. The significance of our findings is confirmed through big-data gene expression studies.

**Supplementary Information:**

The online version contains supplementary material available at 10.1007/s11357-022-00645-w.

## Introduction

Aging elevates the risk of tissue degeneration and metabolic pathologies, perturbs molecular and cellular homeostasis, and leads to global and multiple loss of organ functions [1, 2]. The United Nations reported that life expectancy for the world’s population will reach approximately 77.1 years by 2050, and the number of people above age 80 is expected to triple from 143 million in 2019 to 426 million by 2025 [3]. Our aging world is projected to become socio-economically unsustainable. Thus, it becomes essential to better understand the process of aging and to translate experimentally proven rejuvenative strategies to the clinic.

The deregulation of the immune system with age eventually leads to chronic inflammation, also known as inflammaging [4]. An increase in inflammation is found even in relatively healthy older individuals [4, 5]. Aging of the immune system is also characterized by alterations in the self-renewal and differentiation of hematopoietic stem cells (HSCs), where myeloid blood cells accumulate at the expense of lymphoid cells [6–8]. This causes the impairment of adaptive immunity in older individuals, reducing their ability to combat novel infections and eliminate oncogenically transformed cells. Of note, myeloid skewing increases the risks of age-associated myeloid leukemias in the old, as opposed to pediatric leukemias, which are primarily lymphoid-derived [6, 9]. The many alterations of the immune system with age [10] manifest in the peripheral blood mononuclear cells (PBMCs) including NK cells [11], macrophages [12], and lymphoid lineages [13].

Considering these systemically propagated, age-imposed changes, it is not surprising that blood sharing between young and old mice has rapid and robust pro-geronic and rejuvenative influences [14, 15]. Interestingly, the procedure of small animal plasma exchange to dilute the circulating factors in plasma effectively reset the age-elevated systemic proteome and restored youthful healthy maintenance and repair of muscle, liver, and brain, without any added young blood, young plasma, or young factors [15–17].

For people, plasma dilution is known as plasmapheresis or therapeutic plasma exchange (TPE); it replaces a patient’s plasma with saline and purified albumin. The blood cells are returned to the patient so that while the cell profile does not change, the circulating blood proteins are diluted, including cytokines, autoreactive antibodies or toxins, and such pathogenic determinants of specific disorders [18]. Although its full therapeutic benefits are still being discovered, TPE is one of the treatments for autoimmune and neurological diseases such as myasthenia gravis, Alzheimer’s disease, and Guillain–Barre syndrome [19, 20]. Moreover, TPE has the capacity to relieve the symptoms of long-haul COVID-19, including prevention of pneumonia, reduction of “brain fog,” and attenuation of the cytokine storm and hyper-inflammation [21–23].

Here, we followed the effects of a miniaturized TPE in mice and of pilot studies of TPE with 3 human patients [16, 17] by studying the longitudinal effects of rounds of TPE on hallmarks of systemic aging. The results demonstrate significant and lasting rejuvenation of both humoral and cellular blood compartments in people who underwent repeated plasmapheresis. The rejuvenative changes are not limited to a reduction of inflammaging but encompass diminished circulatory protein markers of neurodegeneration and cancer, as well as reduced senescence, lower DNA damage, and improved myeloid/lymphoid homeostasis. Mechanistically, these and previously reported positive effects of TPE become better understood through longitudinal comparative proteomics of the blood plasma, demonstrating a youthful recalibration of the canonical signaling pathways, broadly regulating tissue health, and interacting through the node of TPR-4. Lastly, a novel application of Levene’s test to profile the noise of the systemic proteome uncovered several proteins: new biomarkers that collectively quantify a person’s biological age, removing a need for predictions.

## Results

### Rounds of TPE diminish DNA damage and senescence of PBMCs

**Fig. 1:**
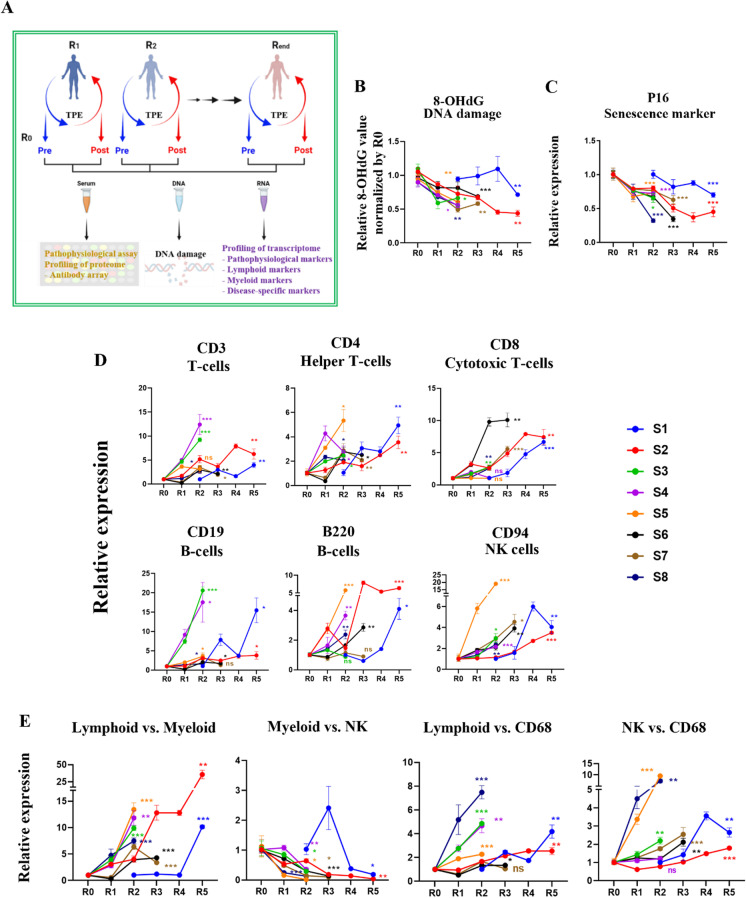
The remodeling effect of TPE treatment on aged blood. **A** Schematic depicting the study; R0 is before TPE, R1 is 1 month afterwards and before the next round of TPE, and so on. **B**. Changes in 8-OHdG levels after TPE. S is the subject or patient number. Oxidative DNA damage gradually decreases and becomes statistically lower by the last round, in all patients. 8-OHdG ELISA was performed on each sample in triplicates. **C** TPE decreases p16 levels in PBMCs of old and middle-aged people, as assayed by qRT-PCR. **D** TPE upregulates the markers of lymphoid genes (T cells, B cells, NK cells) in old PBMCs. **E** The lymphoid:myeloid ratio is increased by the rounds of TPE. The myeloid:NK ratio is downregulated by TPE. The ratios of lymphoid:CD68 and NK:CD68 are elevated by the rounds of TPE. These data show a rejuvenation of the lymphoid/myeloid balance, suggesting an improved capacity for productive immune responses. Each gene profiling is performed by qRT-PCR in 3 replicates for each sample. *p < 0.05, **p < 0.01, ***p < 0.001, ns = not significant

To investigate the effect of TPE, we examined blood samples before and after rounds of this clinical procedure (Fig. 1A and Supplementary Fig. 1). The samples were de-identified and used as per the approved IRB (see “Materials and methods”). Each sample was separated into plasma/serum and cells, as published [21]. Our focus was on assaying the effects of TPE rounds on such hallmarks of aging, as DNA damage and cellular senescence. Samples 1, 2, 4, 6, 7, and 8 were from old individuals (77, 67, 72, 68, 60, and 72 years of age), while samples 3 and 5 were from middle-aged people (46 and 52 years of age) (Supplementary Table 1).

DNA damage can be caused by exogenous and endogenous sources, exemplified by X-rays, UV, and ROS [24, 25]. Accumulated DNA damage triggers genetic aberrations, senescence [26], and loss of cell function and leads to age-related diseases [24].

Oxidative DNA damage was assayed in PBMCs with the 8OH-dg kit (IT7974, G-Biosciences) (Fig. 1B). The relative level of 8-OHdG was high before TPE, and although there is a difference in the fold change among the samples, the DNA damage was significantly decreased by the rounds of TPE (Fig. 1B).

For assaying senescence, messenger RNA (mRNA) was isolated from the PBMCs and profiled with qRT-PCR for p16, using GAPDH as the control (Fig. 1C). Expression of the p16 senescence marker was high in the PBMCs before TPE and was reduced by subsequent rounds of TPE (Fig. 1C).

Tumor-related genes were also profiled by qRT-PCR on PBMCs. There was no significant change in the expression of c-Myc, a known oncogene, while the expression of tumor suppressor genes such as P21 and p53 increased after TPE treatment in most patients (Supplementary Fig. 2).

These results demonstrate that repetitive plasmapheresis reduces the markers of senescence and DNA damage in human PBMCs.

### Rounds of TPE gradually restore youthful lymphoid/myeloid markers to old PBMCs

To determine the effects of repeated TPE on the age-imposed myeloid skewing, we performed qRT-PCR on the gene expression pattern that reflects the lymphoid cell fates and macrophage-linked inflammaging (Fig. 1D). Lymphoid and natural killer cells decrease with aging, while macrophages, and particularly inflammatory CD68 + macrophages, increase [11, 26–28], explaining the age-related deficiency in combatting viral infections and the tendency to easily develop hyper-inflammation [27].

As shown in Fig. 1D and E, rounds of TPE increased the CD3, CD4, and CD8 markers in PBMCs. For the B cell markers, there was a large sample-specific range, but CD19 and B220 were generally induced by the rounds of TPE (Fig. 1D, E). CD94, a NK cell–specific marker, was low in the old PBMCs, but rebounded after the rounds of TPE (Fig. 1D, E).

Further suggesting a rejuvenation of the leukocyte subsets, the expression of macrophage-specific markers, CD11b and CD68, was generally reduced by the rounds of TPE (Fig. 1D, E). Interestingly, these transitions were clearly longitudinal and gradual, becoming more statistically significant with more rounds of the procedure and stably improving overall for several months. And importantly, the positive effects of TPE were maintained for at least 1 month, the time from one round to another. In agreement with the increase in lymphoid gene markers and diminished inflammatory markers, the *ratios* of lymphoid to myeloid markers showed a sharp increase through the rounds of TPE, suggesting that this procedure re-balances adaptive immunity and that it diminishes the cellular signatures of inflammation (Fig. 1E). In addition to the net change in the lymphoid/myeloid markers, which is shown in Fig. 1D and E, the direct comparison between the R0 and Rlast rounds for each marker is shown in Supplementary Fig. 3.

These results demonstrate that repetitive TPE diminishes the pro-inflammatory leukocyte skewing and upregulates the lymphoid markers of old and middle-aged human PBMCs.

### Stable and significant longitudinal rejuvenation of systemic proteome by TPE

**Fig. 2:**
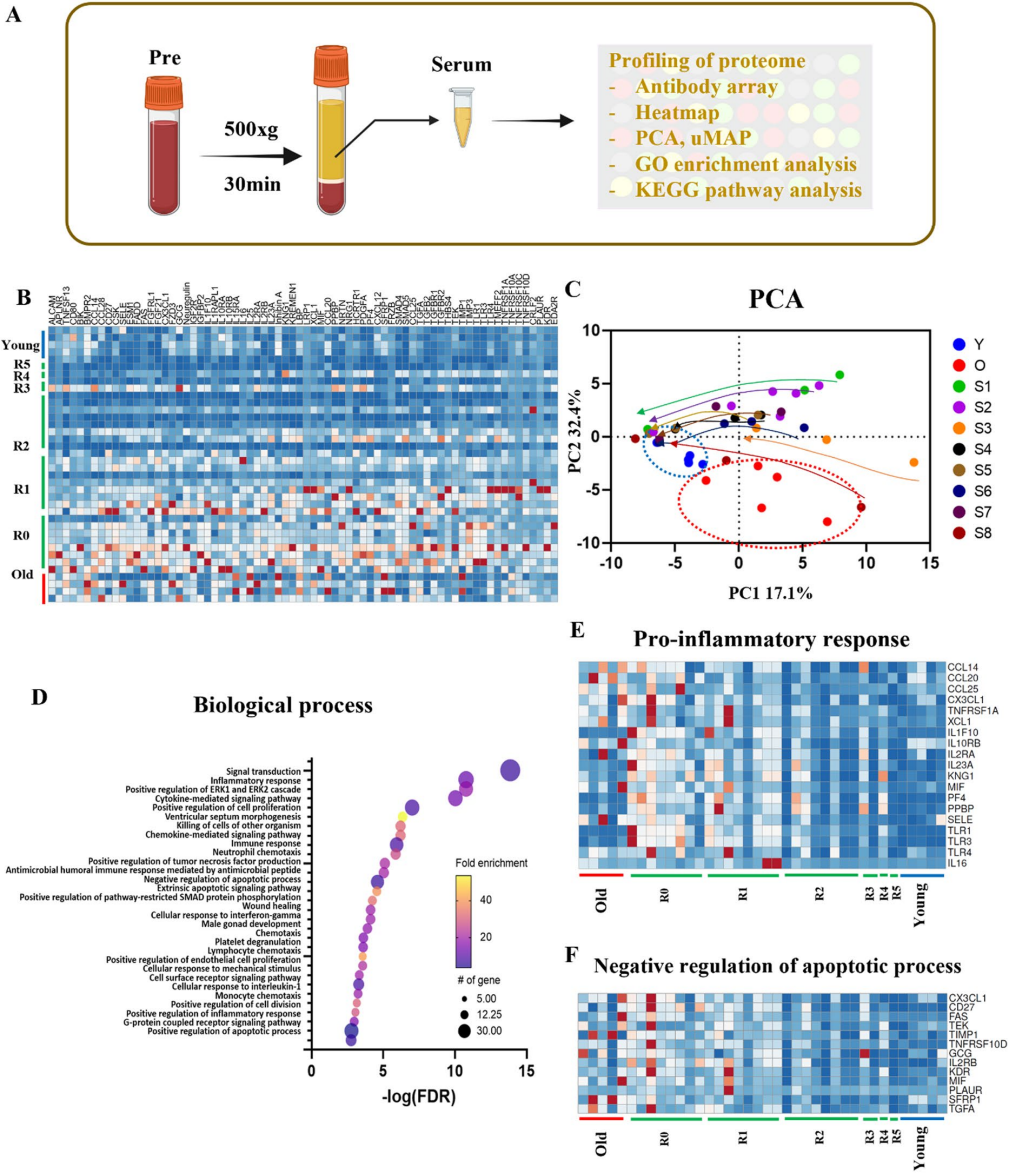
Proteomic profiling of TPE serum using antibody array. **A** Schematic of the study. **B** Heat mapping of the 72 proteins that are significantly different (> 1.75 fold change) between the young and the old cohorts. **C** Principal component analysis (PCA) of the antibody array data of the young, old, and TPE groups for all rounds. Young, *N* = 5; old, *N* = 5; TPE, *N* = 8 (e.g., 8 patients in each TPE round). Arrow tails are R0, and heads are the last round for each group. **D** The top 30 Gene Ontology terms of biological processes. **E** The heat map of inflammatory response protein levels (*p* = 4.10E − 14). **F** The heat map of apoptosis protein levels (*p* = 3.60E − 07). Most proteins in the three different terms were close to old before TPE treatment and became close to the young group over monthly rounds of TPE

Next, we profiled the serum samples from young and old control donors (young, 28–32 years of age; old 70–79 years of age, Supplementary Table 1) and the longitudinal samples from TPE patients, using RayBiotech antibody arrays (Fig. 2A and Supplementary Fig. 4A). Out of 507 proteins, 72 proteins were significantly different in their levels between the old and the young groups (> 1.75 fold change, *p* < 0.05). These 72 proteins were analyzed further in the longitudinal TPE datasets by heat mapping, which revealed a gradual rejuvenation of the age-specific systemic proteome by subsequent rounds of TPE (Fig. 2B and Supplementary Fig. 4B). PCA confirmed that each R0 proteome (before the first TPE) was closer to the old control group than to the young control group and shifted from the old toward the young group with the rounds of TPE (Fig. 2C).

The Gene Ontology (GO) analysis through DAVID (version 6.8, https://david.ncifcrf.gov) with the 72 selected proteins revealed that 226 terms were in biological processes (BPs), 29 terms were related to cellular components, and 40 groups belonged to molecular functions (Fig. 2 and Supplementary Fig. 5).

With respect to BPs, the top 30 GO terms are presented in Fig. 2D and Supplementary Fig. 5, and many of these participate in signal transduction (*p* = 1.50E − 17); regulate the inflammatory response (*p* = 4.10E − 14); positively regulate the ERK/ERK2 cascade (*p* = 5.50E − 14) and the cytokine-mediated signaling pathways (*p* = 4.00E − 13); positively regulate the cell proliferation (*p* = 5.20E − 10), chemokine-mediated signaling pathways (*p* = 5.40E − 09), immune response (*p* = 1.20E − 08), and chemotaxis (*p* = 5.00E − 06); and negatively regulate the apoptotic process (*p* = 3.60E − 07).

The inflammatory response is both an essential defensive system and a hallmark of age-related dysfunctions [29]. We identified 19 proteins which were upregulated before the first TPE treatment or in the control old group, as compared to the young group (Fig. 2E). Notably, many of these proteins, including CCL20, CCL25, MIF, TLR3, TLR4, IL-2RA, and IL-16, were gradually downregulated by the rounds of TPE, with the expression levels measured just before the final round being close to the young levels (Fig. 2E).

Some difference in the levels of specific proteins was observed within the old cohort and between the old and TPE R0 cohorts, which could be explained, for example, by the differences in the ages, lifestyles, genetics, etc., parameters of the donors of commercial blood samples and those who were undergoing the TPE (Fig. 2E).

We also determined whether repetitive TPE may regulate the complement system including C3 and C1q, which play a key role in immune responses and also participate in non-immune crosstalks of cell–cell signaling pathways [30–32]. When we examined 15 commercial human serum samples across young (21–26), middle (46–52), and old (70–74) ages, we found that C1q and C3 levels were significantly elevated with age (Supplementary Fig. 6A). Mean levels of C3 and C1q complement proteins were significantly reduced by roughly half immediately following a TPE procedure, as expected from the immediate dilution of plasma, but on average, they returned to the initial levels by the next round ~ 1 month later. Even three TPE rounds had no significant lasting effects on the serum levels of C3 or C1q (Supplementary Fig. 6B and C).

Lastly, we analyzed the systemic regulators of apoptosis. Apoptosis plays an important role in immune responses and tissue homeostasis [33, 34]. However, with advancing age, resistance to apoptosis is increased through an enhanced negative loop of anti-apoptotic signaling, leading to senescence, inflammation, fibrosis, and a propensity for cancer [35–37]. In our BP analysis, 13 proteins were related to the negative regulation of apoptosis and the levels of these proteins were higher in the old group than in the young (Fig. 2F) [35–37]. Consistent with better tissue homeostasis, the levels of these apoptotic inhibitors diminished over rounds of TPE, becoming closer to the young cohort (Fig. 2F).

Therefore, rounds of TPE gradually reset the circulating protein markers of key cellular responses to their younger levels.

### Pathway interaction String analysis defines the rejuvenation interactome

**Fig. 3:**
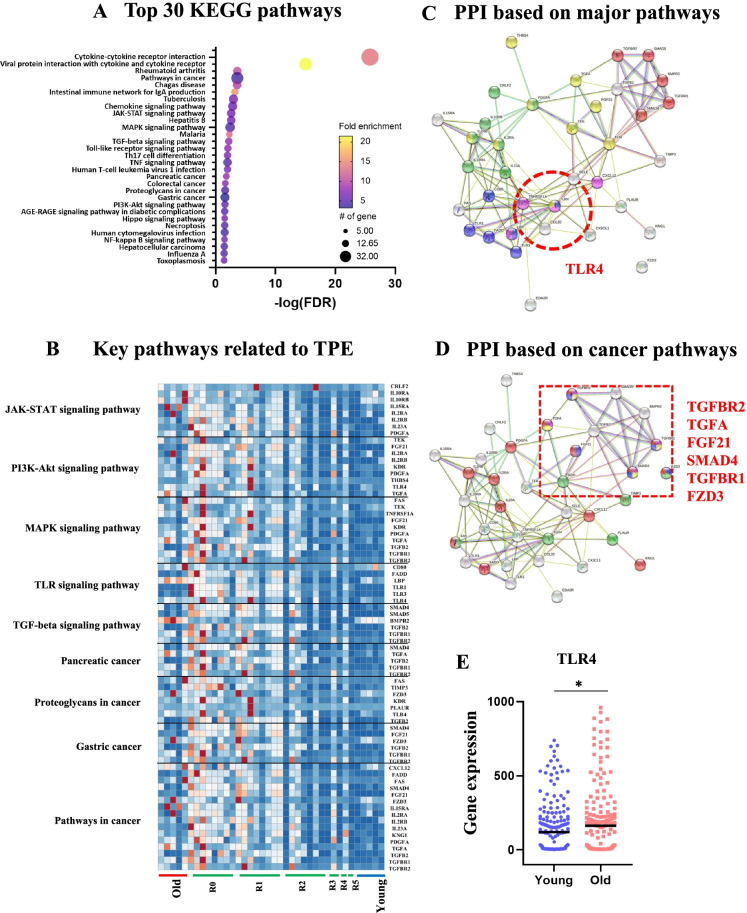
Profiling-relevant mechanisms of TPE using selected 72 proteins. **A** Top 30 KEGG pathways. **B** Heat map with designations of the key functions of the groups of proteins. **C** The protein–protein interaction (PPI) network analysis of the canonical signaling pathways identifies TLR4 as the crosstalk node. **D** The PPI network analysis of the pathways in cancer identifies six designated nodal points of their interaction. **E** TLR4 gene expression levels in 349 young vs. old individuals (120.5 vs. 162.9, *p < 0.05). *N*^young^ = 181, *N*.^old^ = 168

Although TPE treatment has been in clinic for decades, its impact on the process of aging and specifically the links to rejuvenation remain unstudied. To address this limitation, we first applied Kyoto Encyclopedia of Genes and Genomes (KEGG) pathway bioinformatics analysis to the dataset of our comparative proteomics. Forty-three pathways were identified including cytokine–cytokine receptor interaction (*p* = 1.60E − 28), rheumatoid arthritis (*p* = 6.80E − 06), pathways in cancer (*p* = 9.30E − 06), intestinal immune network for IgA production (*p* = 3.50E − 05), JAK-STAT signaling pathway (*p* = 2.40E − 04), MAPK signaling pathway (*p* = 4.00E − 04), TGF-beta signaling pathway (*p* = 7.80E − 04), Toll-like receptor signaling pathway (*p* = 1.20E − 03), and nuclear factor (NF)-κB signaling pathway (*p* = 5.80E − 03) (Fig. 3A).

Interestingly, we also found KEGG terms related to cancer (Fig. 3B), e.g., a disease with a well-known age-elevated risk [38]. There are many reasons as to why cancers occur more frequently with aging, including the decline in productive immune responses, inflammation, the overall aging of immune cells [38], and the accumulation of senescent cells [39]. In agreement with the age-related etiology of cancers, most KEGG cancer terms, including pancreatic cancer, and proteoglycans in cancer were upregulated in the control old groups, as compared to the control young group (Fig. 3B). Notably, these excessive cancer pathway proteins were downregulated by the rounds of TPE (Fig. 3B).

Based on the broad multitissue rejuvenation by the old plasma dilution, we noted those pathways among the 43, which are known to be altered with aging in ways that interfere with the maintenance and repair of multiple tissues: the JAK-STAT [40], MAPK/ERK1/2 [41], TGF-beta [35], NF-κB [42], and Toll-like receptor signaling [43] (Fig. 3B and Supplementary Fig. 7).

To analyze the pathway interactions and the crosstalks between key proteins, we used a protein–protein interaction (PPI) network, the String [44] (Fig. 3C, D). The String consists of known and predicted PPIs including physical and functional associations from many data sources spanning more than 14,000 organisms [44, 45]. Moreover, String allows the display of various functional studies simultaneously with PPIs including GO, KEGG, and InterPro, facilitating the search for nodal proteins [44, 45]. With selected 72 proteins, PPI enrichment *p* values were less than 1.0E − 16, demonstrating that their interactions were significantly meaningful (Fig. 3C, D).

Through String analysis, we found that only one protein (TLR4) was linked to all the age-specific TPE-rejuvenated networks (Fig. 3C). Notably, the levels of TLR4 protein were significantly decreased after TPE, as compared to before this procedure (Supplementary Fig. 8). In addition, String pathway interaction analysis identified six proteins (TGFBR2, TGFA, FGF21, SMAD4, TGFBR1, and FZD3) that were at the intersection of the cancer-associated pathways and, importantly, which were restored to their younger crosstalks by the rounds of TPE (Fig. 3D).

To test and expand the significance of the findings that TLR4 is a nodal point of the age-altered and TPE-normalized pathway interactions, we analyzed the age-specific levels of TLR4 gene expression in 349 individuals (young [20–29 years] and aged [65–75 years]), using data mining of publicly available datasets [46–54].

TLR4 expression increases with age, in agreement with our proteomics and confirming the significance of the long-term attenuation of TLR4 by TPE (Fig. 3E).

These data demonstrate that rounds of TPE normalize the interactions between several key pathways, which, at their young signaling intensity, are known to be responsible for the homeostatic health of multiple organ systems, including the immune system. TLR4 was identified as a potential nodal point of the age-specific TPE-balanced signaling crosstalks.

### Age and neurological disease–specific clustering of systemic proteomes and the reduction of a circulating biomarker of neurodegeneration, TDP43, by TPE

**Fig. 4:**
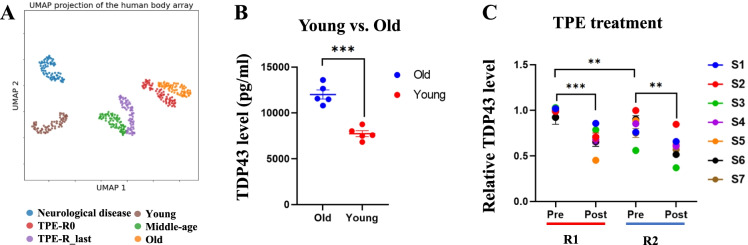
Age and dementia–specific blood proteome clustering and reduction of blood TDP43 by TPE. **A** Distinct UMAP clustering of the 72 proteins that differ between Y and O, for the six age and disease groups showing that the population of the R0 is close to the old group. However, the R last cluster is closer to the middle-aged group. **B** Circulating TDP43 levels are higher in old than in young individuals (12,024 ± 399 vs. 7732 ± 346.8). Values are mean picograms/milliliters ± SEM. **C** TDP43 levels diminished right after TPE (R1: 0.99 ± 0.01 vs. 0.66 ± 0.03, R2: 0.82 ± 0.04 vs. 0.58 ± 0.04) and remained significantly low for 1 month until the next round (0.99 ± 0.01 vs. 0.82 ± 0.04). **p < 0.01, ***p < 0.001

Aging is accompanied by various diseases with neurological disorders being a prominent class [55]. The blood biomarkers of brain diseases remain unknown, but promisingly, the Uniform Manifold Approximation and Projection (UMAP) analysis of our comparative blood proteomics defined distinct clustering of old people with Alzheimer’s disease (AD) and AD-related diseases (ADRDs), as compared to the healthy age-matched and younger cohorts (Fig. 4A and Supplementary Table 1). In agreement with the data on rejuvenation of the systemic proteome which we described above, the rounds of TPE moved the old blood proteome cluster of this AD/ADRD dataset toward the younger age cohort (Fig. 4A). Further substantiating the robustness of our results, the healthy old proteome of the age-matched controls of the AD/ADRD samples clustered close to the pre-TPE proteome, an independent dataset of healthy old blood donors (Fig. 4A).

Considering these observations, we decided to analyze systemic levels of TDP43, which is the trigger of several neurological pathologies and becomes increased in the blood of patients with ALS, Parkinson’s disease (PD), frontotemporal dementia (FTD), and AD [56–61].

We analyzed the levels of this protein in young, old, pre-TPE, and after TPE serum samples. Interestingly, a TDP43-specific enzyme-linked immunosorbent assay (ELISA) demonstrated a robust age-specific increase in this determinant of neurological diseases in the serum of old, relatively healthy adults, as compared to the young cohort (Fig. 4B). Moreover, the longitudinal studies on the plasma before vs. after the rounds of TPE demonstrated that systemic TDP43 was stably attenuated by plasmapheresis (Fig. 4C). Notably, the overall levels of TDP43 in older adults were not just transiently diluted by the procedure, which is expected, but remained lower for at least a month after TPE (Fig. 4C).

These results suggest that the selected proteins enable the identification of AD/ADRD through UMAP analysis of blood plasma and confirm that TPE promotes a younger systemic proteome. Additionally, we show that TPE stably attenuates a known biomarker of neurological diseases, TDP43.

### A novel biomarker of protein level noise provides a direct molecular measurement of human biological age and shows that it is reduced by TPE

Biological noise is the variation of a given biological parameter in an otherwise assumed constant or steady state. Several papers suggest that biological noise increases with age [62, 63], and noise of gene expression and protein levels is an independent age-related parameter that is relatively less studied, as compared with total levels of the genes, proteins, and other cellular and tissue metrics. An unmet need remains in identifying reliable molecular biomarkers that allow *measuring* biological age, in contrast to predicting it by data selection and adjustment, such as machine learning models of population statistics. We approached this problem by comparing not just young vs. old samples, as typically done, but also the rejuvenated cohort, and moreover, in the longitudinal studies of the same older individuals whose blood compartments became more like those of young people after TPE, based on the cellular and humoral blood assays.

To test if not just the protein levels but also their noise became more youthful through TPE, we compared the standard deviations (SDs) of protein levels, using our comparative proteomics datasets: the control young and old cohorts, round 0 vs. the last round of TPE, and of the old cohort with the cognitive disorders (progressive AD and ADRD). As the measurement uncertainty is similar between populations, any change in SD should reflect biological variation; here, biological variation is the levels of protein expression. A change in this variation could mean many things, from a loss of transcription regulation to stochasticity in protein quality checking to changes in turnover, but the end result is that protein levels are either more heavily *controlled* for lower SD or less heavily controlled for a greater SD.

**Fig. 5:**
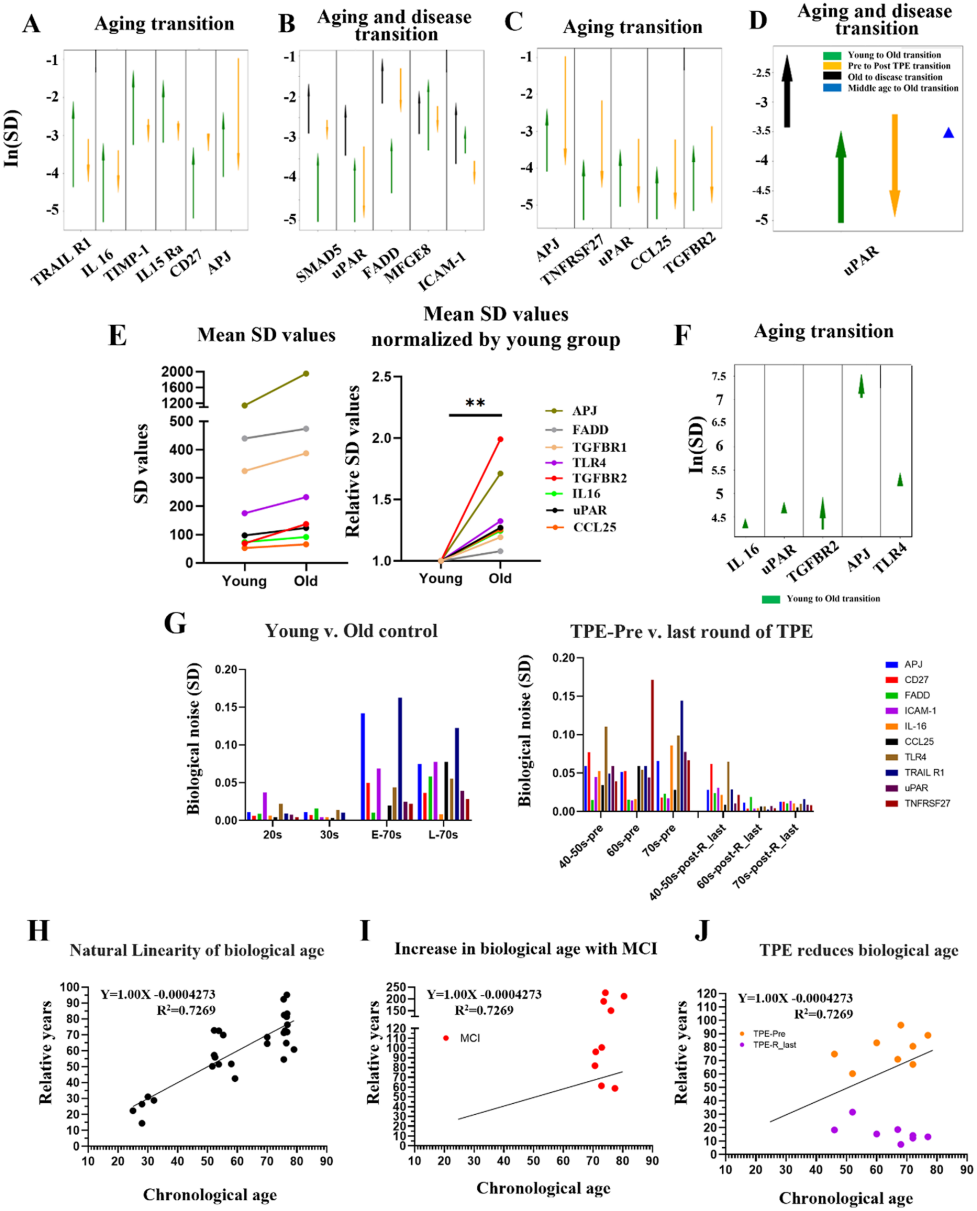
Profiling changes in biological noise using standard deviation (SD) among young, old, TPE, and disease cohorts. **A** The change in the SD of the 507 proteins (antibody array proteomics) between young and old people and between people before TPE and people after TPE, in proteins which had a Benjamini–Hochberg procedure false discovery rate of 10% regarding the significance of variance changes with age. **B** The change in SD between young and old people, between people with a neurodegenerative disorder and healthy people of the same age, and between people before and after TPE, in proteins which have significantly different variances in the aging and disease comparisons. **C** The change in SD between young and old people and between people before TPE and people after TPE, in proteins which had significantly different variances between age groups and which had their SD change by a factor of 5 or greater with TPE treatment. **D** The change in SD between young and old people, between people with a neurodegenerative disorder and healthy people of the same age, between people before TPE and people after TPE, and between middle-aged and old people, in proteins which have significantly different variances in the aging and disease comparisons and which had their SD change by a factor of 5 or greater with TPE treatment. In each case, significance was determined with the mean based Levene’s test. **E** Comparison of mean SDs of the mRNA levels of 8 genes between young and old groups, for each SD value of each gene (left), expressed as a fold increase of young (right, **p = 0.003). **F** The change in SD of the 5 genes between young and old people, in proteins which had a Benjamini–Hochberg procedure (see “Materials and methods”) false discovery rate of 10% regarding the significance of variance changes with age. **G** The biological noise was calculated using the SD of 10 selected proteins, divided by age. The biological noise of 10 proteins increases in the old group, compared with the young group. Interestingly, there is a clear and significant decrease of protein noise levels after rounds of TPE for all noise-detectors. **H** Plot of biological age calculated as the SD of the uncovered noise detectors versus chronological age. **I** The distribution of MCI, based on the 10 noise biomarkers. **J** Biological age shifts after repeated TPE treatment. Compared to before TPE treatment, all patients show a decrease in biological age in the last round of TPE, demonstrating significant rejuvenation by TPE

To find statistically significant changes in biological noise, we looked at the difference in variance with Levene’s test. Levene’s test has various possible shortcomings in both power and significance when dealing with small sample sizes and asymmetric distributions [64]. To deal with this, we increased significance through the Benjamini–Hochberg procedure, selecting only proteins with significant changes in multiple populations and selecting proteins with other measures of substantial change in variance, albeit only larger sample size ultimately increases power. We found only 6 proteins showing increased SD in the young-to-old transition and decreased SD in the pre-to-after TPE transition: TRAIL R1, IL-16, TIMP-1, IL-15R alpha, CD27, and APJ (Fig. 5A). Interestingly, when we considered proteins which were significantly different in their SD between the healthy old and the old with cognitive disease and between the healthy young and healthy old, there were just 4 such proteins: Smad5, uPAR, FADD, and TGFBR1 (Fig. 5B). Namely for these 4 proteins, the SD increased in a young-to-old transition and in an old-to-disease transition but decreased in the R0-to-Rlast TPE transition (Fig. 5B).

We next profiled the proteins which had not just a statistically significant change in the young-to-old transition, but also had a substantial change (greater than fivefold) in SD with TPE. We found 5 such proteins: APJ, TNFRSF27, uPAR, CCL25, and TGFBR2 (Fig. 5C). Very interestingly, when the field was further narrowed to only those proteins which also have significant SD changes in the healthy-to-disease transition, there was only one protein showing increased SD in young-to-old transition and old-to-disease transition, but decreased SD in pre-to-post-TPE transition, uPAR (Fig. 5D). And for this best noise detector protein, the middle-aged individuals lay right between the young and old individuals (Fig. 5D).

To confirm and extrapolate the significance of the identified protein determinants of age-related biological noise, their mRNA expression was analyzed in 349 individuals from publicly available datasets [46–54]. Interestingly and in agreement with our comparative proteomics data, the mean SD values, e.g., the noise, of all these genes increased with age, albeit to a different degree for the different transcripts (Fig. 5E).

Their mRNA levels were also age-elevated (Fig. 3E [for TLR4] and Supplementary Fig. 9 [for other transcripts]). These results agree with the proteomics data and expand the significance of TLR4 as a nodal point of the age-altered, signal transduction networks (Fig. 3B, C).

Supplementary Fig. 10 arranges the data by the size of SD of protein levels in a healthy population, which gives further credence to the idea that the changes in SD are the consequence of the age/disease-imposed biological noise. Namely, the proteins that are most tightly controlled initially were also those most likely to have a significant change in SD (deregulation) with age and disease. Supplementary Fig. 10 also shows that TPE is indeed returning the SDs to their younger lower states, e.g., the noise dampening effects of TPE are manifested more on the proteins that become less controlled or noisier with age. Of note, there is no uniform change in variance in noise with age; instead, those proteins which are least controlled change most and vice versa.

Our next goal was to apply the discovered biomarkers of proteome noise toward a novel measurement of person’s biological age. There is an unmet need for unambiguous molecular quantification of biological age based on experimentally defined biomarkers, without machine learning (ML) methods, large data statistics, and data adjustments and with improved resilience to batch effects [65, 66]. Typical ML models are trained on chronological age and predict chronological age. The connection of a correlate (DNA methylation, protein levels, etc.) to biology is not straightforward, and in fact, the experimental data is numerically adjusted (variably for different data points) to correlate with the chronological age better linearly. Large datasets are used, but each line has *N* = 1 with a lack of verification.

The age-increased and TPE-reduced changes in the SD of the 10 protein biomarkers (Fig. 5F) suggested the possibility to uncover a natural linearity between the chronological age and the biological age, through these noise reporters. Although the mean protein levels tend to increase with age, variance also often increases. Namely, in some old samples, the levels will be similar to the young samples, and in other old samples, they will be significantly different, which is reflected in high SD. To test if the 10 protein biomarkers would enable a direct quantification of person’s biological age, we calculated the mean of their SDs (10-protein noise) and plotted these values vs. chronological age of the young (20–30 years), middle-aged (50–60), and old (70 +) individuals (Fig. 5G). Such direct analysis yielded a linear measure of biological age, which we define as an increase in the 10-protein noise (SD) with a Pearson’s *R* value of 0.7269 and a *p* value of < 0.0001 (Fig. 5G).

Next, we tested if TPE reduces the directly quantified biological age and if brain dysfunction (mild cognitive impairment, MCI) increases the biological age of chronologically similar people. As shown in Fig. 5H, based on the 10-protein noise, the biological age of people with MCI was increased by over 50 years (the average of biological age = 130.9 years), while the biological age of 40–70 + individuals became reduced by decades after multiple rounds of TPE (Fig. 5I). The additional analysis of the relative biological noise age via TPE treatment demonstrated that it becomes reduced more sharply in individuals aged 60–70 + years, as compared to people who are 46–52 years old (Supplementary Fig. 11).

These results establish that person’s biological age can be not just predicted from large datasets, but directly and accurately measured by the combined 10-protien noise; these direct quantifications demonstrate a natural linearity of the 10-protein noise with chronological age, a reduction of such biological age by three rounds of TPE, and an increase by MCI. These results additionally suggest that age and neurologic disease increase the noise of the systemic proteome, most noticeably when looking at uPAR, TRAIL R1, IL-16, TIMP-1, IL-15R alpha, CD27, and APJ, TNFRSF27, CCL25, and TGFBR2. These conclusions are derived from comparative proteomics on over 50 samples and are confirmed through the gene expression analyses of 349 individuals.

## Discussion

This study establishes the self-sufficiency of the positive effects of plasma dilution, in the absence of young blood or its factors, for stable rejuvenation of humoral and cellular blood compartments in people. Specifically, repetitive TPE reduces the age-imposed skewing toward myeloid PBMC markers, which is closely linked to inflammaging [11, 26, 27] and restores the balance of lymphoid cell markers to the old PBMCs. TPE simultaneously ameliorates the hallmark pathophysiological changes, such as oxidative DNA damage, senescence, systemic markers of cancer pathways, and neurodegeneration-related circulating TDP43.

Moreover, comparing not just the young and old samples, but those who were longitudinally treated and rejuvenated, allowed us to uncover 10 novel protein biomarkers that report biological noise and enable the direct measurement of biological age. The natural linearity of biological age is remarkable and the biological significance of the 10 novel protein biomarkers is known: they become noisy with age and MCI and less noisy after a treatment that promotes broad rejuvenation of humoral and cellular blood compartments in people. Future studies will delineate the mechanisms by which these 10 proteins become deregulated with age and how this affects cells and tissues. While overall multiparametric biological age of an organism is expected to be different from that of the humoral and cellular blood compartments, rejuvenation of the blood is expected to manifest in many organ systems. As expected, many other proteins did not show an age-related increase in their SD and some showed an age-imposed decline in the SD, which might indicate lower complexities of old tissues and would be interesting to address in future work.

In this study, we found that proteomic noise increases with age and discovered candidates for specific biomarkers for aging and disease through their high variability. In support of the robustness of our conclusions, our comparative proteomics fits well with independent large gene expression datasets and is consistent with the notion that biological noise increases with age [62, 63, 67]. Interestingly, the proteins with the highest SD became the most controlled proteins after TPE treatment, becoming the lowest-noise cohort. Summarily, our results suggest that TPE facilitates the reduction of age-imposed biological noise, and this effect may be related to immune system, inflammatory response, and the various cellular pathways that we delineated above. Additionally, the 10-biomarker noise quantification and UMAP distinguish between people who are healthy vs. those who have AD/ADRD and show promise for definitive blood biomarkers of brain diseases.

Some of the phenomena studied here are linked; for example, DNA damage [24, 25] affects not only cell health but also cell fate, leading to a loss of tissue homeostasis, chronic inflammation, and other dysfunctions [24, 68]. In particular, immune cells with damaged DNA interfere with productive immune responses and with the homeostatic immunity–inflammation axis and cause inflammaging [69]. The cell fate changes during hematopoiesis trigger lymphoid/myeloid skewing and result in inefficient immunity [4, 69, 70]. When the immunity–inflammation axis is imbalanced, autoimmune diseases, neurodegenerative diseases, or cancer have higher risks [29, 71, 72]. Thus, the younger lymphoid/myeloid balance that is promoted by TPE in aged PBMCs and the lower DNA damage and senescence observed in these cells might be linked or synergistic; all have implications for improving immune responses and avoiding or attenuating age-related flares of infections and cancers.

In agreement with an age-imposed increase in cancers [73], our data shows that the levels of many cancer-related proteins were high in the aged blood before TPE. Although some tumor suppressors were identified by the KEGG analysis of our comparative proteomics, e.g., TIMP3 and SMAD4 [74, 75], most proteins of the cancer group in our dataset were oncogenic. For example, SDF-1 is expressed in various cancers, such as ovarian cancer, breast cancer, thyroid cancer, and lung cancer [76–79], and contributes to metastasis [76]. TGF-alpha plays an important role in promoting carcinogenesis and progression of malignancy [80, 81]. The expression of KDR contributes to cancer development through vascularity and is associated with metastasis in colon and breast cancers [82, 83]. Notably, the age-increased cancer-related factors including the above proteins were downregulated after the rounds of TPE. The PPI network analysis with cancer-related terms defined six proteins: TGFBR2, TGF-alpha, FGF21, SMAD4, TGFBR1, and FZD3, most of which are involved in tumor development [84–86]. Moreover, in profiling the PBMCs, we found that the tumor suppressors p21 and p53 were increased by TPE, but there was no change in the expression of the oncogene c-Myc. Overall, these data suggest that TPE might overall decrease the propensity for cancer.

The mechanism of the rejuvenating effects of repetitive TPE is suggested by the results of the longitudinal comparative proteomics, which identifies the key interactive signaling crosstalks and TLR4 as their node. Moreover, gene expression analysis of the larger datasets confirms that the levels of TLR4 increase with age, emphasizing the significance of its TPE-related attenuation. TLR4 participates in the production of inflammatory cytokines through NF-KB signaling [87], but has many other pleiotropic activities [87–89], and the expression of TLR4 is elevated in the aged brain, which might contribute to the development of neurodegenerative disorders [90]. The effects of the age-specific pathways on tissue maintenance and repair, senescence, apoptosis, DNA repair, and cell fate determinations in the blood lineages are well known [40, 91–100], and we show that this pivotal network is reset by TPE to a younger state. Of note, each of the age-specific TPE-influenced pathways is morphogenic and multifunctional and exerts different effects based on their intensity and the overall signaling landscape. Thus, it is important that our study identified *the specific proteins* belonging to these terms that were deregulated in the aged blood and became gradually yet stably normalized by the rounds of TPE, in correlation with the rejuvenated PBMCs.

TPE was tested for treating the AD, as the pathogenic amyloid-β (Aβ) peptides circulate in the blood and bind to serum albumin [101], and removing this peripheral Aβ peptides might allow more brain Aβ peptides to be cleared to the blood. While no statistical significance was found, there was a trend in delaying the progression of early AD through TPE [102]. However, the mechanistic connection between TPE and neurological diseases is largely unexplored [103] in general, or in relation to the diminished brain fog of long COVID-19 [21, 22, 104].

In the current work, we show that TDP43 is elevated in the serum of relatively healthy older adults and is stably attenuated by the rounds of TPE. These data add to previously published reports that TDP43 is a key trigger of various neurological diseases [105] and becomes elevated in the cerebrospinal fluid and plasma of patients with age-associated neurodegenerative disorders [60, 61, 106]. Attenuation of TDP43 by rounds of TPE in undiagnosed adults additionally suggests a preventive capacity against age-associated neurological diseases.

**Fig. 6:**
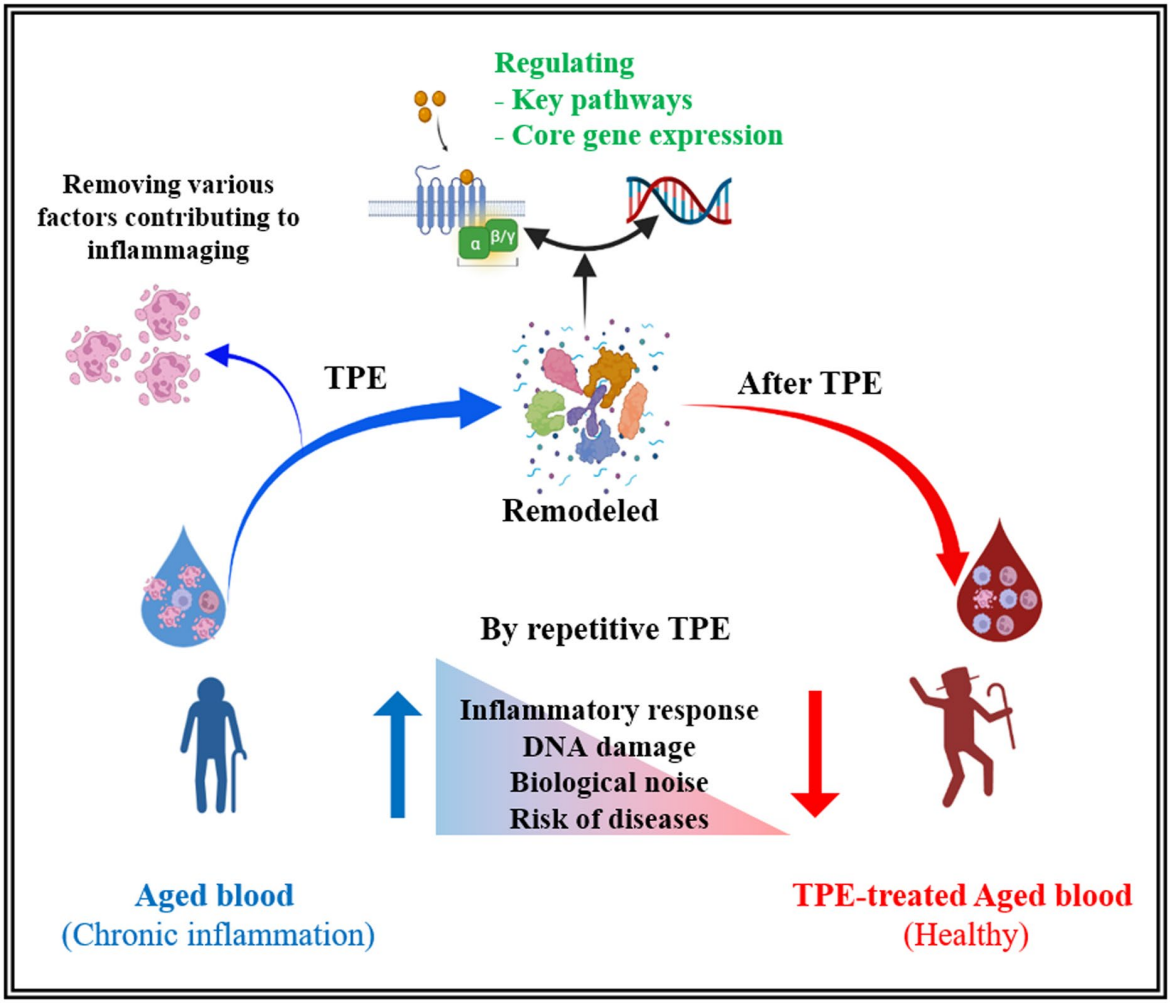
The effect of repetitive TPE on aged blood. Aged blood has the characteristics of chronic inflammation (inflammaging), increased PBMC DNA damage and senescence, and immune deregulation, all changes that promote the high risk of diseases. TPE resets the systemic milieu to a younger state by rapidly and significantly diluting the age-elevated inhibitors of the canonical signaling pathways that regulate tissue maintenance and repair. The immediate effect is reduction of inflammation, and the subsequent effects are improvement in cellular and molecular homeostasis from calibrated cell–cell signaling crosstalks and reduction of biological noise that is defined in this study. Longitudinal studies allowed us to reveal novel broadly rejuvenative aspects of this seemingly simple procedure and to derive statistically significant conclusions with less samples than those needed in bulk comparisons. Created with BioRender.com

The graphic summary of our findings is provided in Fig. 6. Of note, we studied not the immediate result of the physical plasma dilution, but the lasting effects from one procedure to another and through the rounds of TPE, spanning up to 12 months, e.g., the stable rejuvenation of the human blood compartments.

## Materials and methods

### TPE

TPE was performed as described [20, 21], using centrifugal blood cell separator (Spectra Optia; Terumo BCT, Lakewood, CO, USA). One plasma volume was removed and replaced with 5% albumin. Each procedure was followed by infusion of 2 g, 10% intravenous immunoglobulin G (IVIG) (Octagam 10%; Octapharma, Hoboken, NJ, USA).

### Serum, plasma, and PBMC isolation

Whole blood (Supplementary Table 1, which shows the ages and genders of all subjects of these studies) was collected using immediately before (pre) or after (post) TPE procedure in the clinic [20]. Then, whole blood in a serum separation tube (02–683-96, BD) was transported to the laboratory within 1–2 h at 4 °C. Before collecting, the tube was warmed in a 37 °C water bath for 10 min and then centrifuged at 510 RCF for 30 min, and serum was collected and then stored at − 80 °C until use. For plasma and PBMCs, 8 ml whole blood was added on top of 3 ml Histopaque (1077, MilliporeSigma) in a 15-ml tube and was centrifuged at 510 RCF for 30 min. After that, whole blood with Histopaque was separated several layers. Plasma was collected and aliquoted until use. Plasma was then stored at − 80 °C. PBMCs were collected directly from between Histopaque and plasma layers, then washed twice with PBS.

### Real-time polymerase chain reaction

Total RNA was extracted from PBMC using the RNeasy Mini Kit (Qiagen), and the SuperScript III First‐Strand Synthesis System (Invitrogen) was used to synthesize cDNA according to the manufacturer’s instructions. Real‐time polymerase chain reaction (PCR) was performed on a Bio‐Rad iQ5 real‐time PCR machine. The primers used for PCR are listed in Table 1 [107–115].

**Table 1:** Primer sequences for real-time polymerase chain reaction

Group	Gene name		Sequence
T cells	CD3	Forward	GATGCAGTCGGGCACTCACT
		Reverse	CATTACCATCTTGCCCCCAA
Helper T cells	CD4	Forward	GCCAACCCAAGTGACTCTGT
		Reverse	TCTCCTGGACCACTCCATTC
Cytotoxic T cells	CD8	Forward	ACTTGTGGGGTCCTTCTCCT
		Reverse	GTCTCCCGATTTGACCACAG
NK cells	CD94	Forward	GAGCCAGCATTTACTCCAGGAC
		Reverse	GCACAGAGATGCCGACTTTCGT
B cells	B220	Forward	ACA GCC AGC ACC TTT CCT AC
		Reverse	GTG CAG GTA AGG CAG CAG A
	CD19	Forward	AAGGGGCCTAAGTCATTGCT
		Reverse	CAGCAGCCAGTGCCATAGTA
Myeloid cells	CD11b	Forward	CAGCCTTTGACCTTATGTCATGG
		Reverse	CCTGTGCTGTAGTCGCACT
	CD68	Forward	GCTACATGGCGGTGGAGTACAA
		Reverse	ATGATGAGAGGCAGCAAGATGG
Oncogene	c-Myc	Forward	ACCACCAGCAGCGACTCTGA
		Reverse	TGCCTCTTCTCCACAGACACC
Tumor suppressor	P21	Forward	GGAAGACCATGTGGACCTGT
		Reverse	GGCGTTTGGAGTGGTAGAAA
Tumor suppressor	P53	Forward	AGGCCTTGGAACTCAAGGAT
		Reverse	TGAGTCAGGCCCTTCTGTCT
Senescence	P16	Forward	TGAGCACTCACGCCCTAAGC
		Reverse	TAGCAGTGTGACTCAAGAGAAGCC
Housekeeping	β-Actin	Forward	TGAAGTGTGACGTGGACATC
		Reverse	GGAGGAGCAATGATCTTGAT

### ELISA

ELISA was analyzed for 8-OHdG (IT7974, G-Biosciences), C3 (ab108822, Abcam), and C1q (ab170246, Abcam), and the sample dilution was performed according to the manufacturer’s protocol (1:800 or 1:100,000). The stained plate was read at 450 nm as well as 570 nm, and each sample was run in triplicate on a SpectraMax iD3 Multi‐Mode Microplate Reader.

### Antibody array

Serum samples from whole blood were analyzed on a human L507 antibody capture array (AAH-BLG-1–4, RayBiotech), processed according to the manufacturer’s protocol. The array slides were imaged by a Molecular Devices 4000b scanner, and data were calculated by GenePix. Array normalization was by the built-in positive and negative controls.

### Bioinformatics analysis

The gene ID of differently regulated proteins was performed using DAVID Bioinformatics Resources (version 6.8, https://david.ncifcrf.gov), as well as the GO analysis of the differential proteins based on the BP, molecular function (MF), and cellular component (CC), and KEGG pathway. Heat maps were performed with ClustVis [116]. String was used for presenting PPIs and key proteins [44].

### Analysis of protein SD

All analyses of protein SD change were done from the antibody array data after normalization of the values by the internal positive and negative controls, using python, specifically the *scipy.stats* library. For this analysis, the significance of SD changes between populations first had to be gauged. This was done with Levene’s test. Levene’s test is used to test the null hypothesis that the variance of population 1 is equal to the variance of population 2. This test is rigorous against type 1 error for normal and non-normal distributions, but is only rigorous against type 1 errors when testing on symmetric distributions [62]. The distribution of protein expression in each of our populations is not known, so we cannot claim with full confidence that our analysis is resistant to type 1 error. It would have been preferential to use the generally more rigorous median and 20% trimmed mean Brown–Forsythe tests, but these tests suffer from extremely low power when dealing with small sample sizes as we have here [117]. To add more rigor against type 1 error, we only considered proteins which passed tests for significance in more than one comparison or which passed another test for type 1 error such as BH or a proxy for significance such as having a substantial SD change. We only compared paired observations for which Levene’s test is valid. From the library obtained through Levene’s test analysis, we selected nine proteins with greater SD in the old group than in the young group and one additional protein (TLR4) was selected through ontology and PPI analyses. To calculate the biological age, we quantified the mean of the SDs of the 10 proteins and plotted these values vs. chronological age; the trendline and Pearson’s *R* between the noise and chronological age were calculated by GraphPad Prism software version 9 (linear regression analysis). Then, the biological age was calculated using the equation of the trendline.

### Gene expression profiling and availability

The gene expression datasets can be found on the Gene Expression Omnibus website under the following accession numbers: GSE362, GSE674, GSE58015, GSE32719, GSE38718, GSE9103, GSE25941, GSE53890, GSE21779, GSE28422, and GSE28392. We used only young (20–29-year-old) and aged (65–75-year-old) cohorts (TLR4: *N*^young^ = 181 and *N*^old^ = 168, CCL25: *N*^young^ = 181 and *N*^old^ = 168, IL-6: *N*^young^ = 181 and *N*^old^ = 168, TGBR1: *N*^young^ = 144 and *N*^old^ = 156, TGBR2: *N*^young^ = 183 and *N*^old^ = 158, uPAR: *N*^young^ = 165 and *N*^old^ = 163, FADD: *N*^young^ = 176 and *N*^old^ = 187, APJ: *N*^young^ = 104 and *N*^old^ = 153).

### Statistical analysis

All statistical analyses were performed using GraphPad Prism software version 9. All values are expressed as means ± SEM for independent experiments or SD for replicates. To determine the significance of differences among groups, comparisons were made using Student’s *t* test. The *p* < 0.05 was considered significant.

### Supplementary Information

Below is the link to the electronic supplementary material.

Supplementary file1 (DOCX 2094 KB)

Supplementary file2 (XLSX 10 KB)

## References

[1] Harman D (1994). Age.

[2] Harman D (2001). Ann N Y Acad Sci.

[3] United Nations Department of Economic Social Affairs (2019). World population prospects 2019: highlights.

[4] Ferrucci L, Semba RD, Guralnik JM, Ershler WB, Bandinelli S, Patel KV, Sun K, Woodman RC, Andrews NC, Cotter RJ, Ganz T, Nemeth E, Longo DL (2010). Blood.

[5] Newman AB (2016). Int J Epidemiol.

[6] Kim MJ, Kim MH, Kim SA, Chang JS (2008). Int J Stem Cells.

[7] Geiger H, Rudolph KL (2009). Aging in the lympho-hematopoietic stem cell compartment. Trends Immunol.

[8] Ho YH, Méndez-Ferrer S (2020). Microenvironmental contributions to hematopoietic stem cell aging. Haematologica.

[9] Cho RH, Sieburg HB, Muller-Sieburg CE (2008). Blood.

[10] Castelo-Branco C, Soveral I (2014). Gynecol Endocrinol.

[11] Hazeldine J, Lord JM (2013). Ageing Res Rev.

[12] van Duin D, Mohanty S, Thomas V, Ginter S, Montgomery RR, Fikrig E, Allore HG, Medzhitov R, Shaw AC (2007). J Immunol.

[13] Ademokun A, Wu YC, Dunn-Walters D (2010). Biogerontology.

[14] Conboy IM, Conboy MJ, Wagers AJ, Girma ER, Weissman IL, Rando TA (2005). Nature.

[15] Rebo J, Mehdipour M, Gathwala R, Causey K, Liu Y, Conboy MJ, Conboy IM (2016). Nat Commun.

[16] Mehdipour M, Skinner C, Wong N, Lieb M, Liu C, Etienne J, Kato C, Kiprov D, Conboy MJ, Conboy IM (2020). Aging.

[17] Mehdipour M, Mehdipour T, Skinner CM, Wong N, Liu C, Chen CC, Jeon OH, Zuo Y, Conboy MJ, Conboy IM (2021). GeroScience.

[18] Yilmaz AA, Can ÖS, Oral M, Unal N, Ayyildiz E, Ilhan O, Tulunay M (2011). Transfus Apher Sci.

[19] Kumar R, Birinder SP, Gupta S, Singh G, Kaur A (2015). Indian J Crit Care Med.

[20] Boada M, López OL, Olazarán J, Núñez L, Pfeffer M, Paricio M, Lorites J, Piñol-Ripoll G, Gámez JE, Anaya F, Kiprov D, Lima J, Grifols C, Torres M, Costa M, Bozzo J, Szczepiorkowski ZM, Hendrix S, Páez A (2020). Alzheimers Dement.

[21] Kiprov DD, Herskowitz A, Kim D, Lieb M, Liu C, Watanabe E, Hoffman JC, Rohe R, Conboy MJ, Conboy IM (2021). F1000Research.

[22] Jaiswal V, Nasa P, Raouf M, Gupta M, Dewedar H, Mohammad H, Al Rais Z, Ali Baqer M, Alsabbah A, Ibrahim Y, Salem M, Shammass D, Marashi M (2021). Int J Infect Dis.

[23] Keith P, Day M, Choe C, Perkins L, Moyer L, Hays E, French M, Hewitt K, Gravel G, Guffey A, Scott LK (2020). SAGE Open Med Case Rep.

[24] Schumacher B, Pothof J, Vijg J, Hoeijmakers JHJ (2021). Nature.

[25] López-Otín C, Blasco MA, Partridge L, Serrano M, Kroemer G (2013). Cell.

[26] Linton PJ, Dorshkind K (2004). Nat Immunol.

[27] Drijvers JM, Sharpe AH, Haigis MC (2020). eLife.

[28] Abel AM, Yang C, Thakar MS, Malarkannan S (1869). Front Immunol.

[29] Licastro F, Candore G, Lio D, Porcellini E, Colonna-Romano G, Franceschi C, Caruso C (2005). Immun Ageing.

[30] Naito AT, Sumida T, Nomura S, Liu ML, Higo T, Nakagawa A, Okada K, Sakai T, Hashimoto A, Hara Y, Shimizu I, Zhu W, Toko H, Katada A, Akazawa H, Oka T, Lee JK, Minamino T, Nagai T, Walsh K, Kikuchi A, Matsumoto M, Botto M, Shiojima I, Komuro I (2012). Cell.

[31] Peterson SL, Nguyen HX, Mendez OA, Anderson AJ (2017). Sci Rep.

[32] Peterson SL, Anderson AJ (2014). Exp Neurol.

[33] Ravichandran KS, Lorenz U (2007). Nat Rev Immunol.

[34] Yang Y, Jiang G, Zhang P, Fan J (2015). Mil Med Res.

[35] Bye N, Zieba M, Wreford NG, Nichols NR (2001). Neuroscience.

[36] Gupta S, Agrawal A, Agrawal S, Su H, Gollapudi S (2006). Immun Ageing.

[37] Salminen A, Ojala J, Kaarniranta K (2011). Cell Mol Life Sci.

[38] White MC, Holman DM, Boehm JE, Peipins LA, Grossman M (2014). S Jane Henley. Am J Prev Med.

[39] Campisi J (2013). Annu Rev Physiol.

[40] Price FD, Von Maltzahn J, Bentzinger CF, Dumont NA, Yin H, Chang NC, Wilson DH, Frenette J, Rudnicki MA (2014). Nat Med.

[41] Lin AW, Barradas M, Stone JC, van Aelst L, Serrano M, Lowe SW (1998). Genes Dev.

[42] Adler AS, Sinha S, Kawahara TLA, Zhang JY, Segal E, Chang HY (2007). Genes Dev.

[43] Liu H, Chu S, Wu Z (2021). Immun Ageing.

[44] Franceschini A, Szklarczyk D, Frankild S, Kuhn M, Simonovic M, Roth A, Lin J, Minguez P, Bork P, von Mering C, Jensen LJ (2013). Nucleic Acids Res.

[45] Szklarczyk D, Gable AL, Nastou KC, Lyon D, Kirsch R, Pyysalo S, Doncheva NT, Legeay M, Fang T, Bork P, Jensen LJ, von Mering C (2021). Nucleic Acids Res.

[46] Lanza IR, Short DK, Short KR, Raghavakaimal S, Basu R, Joyner MJ, McConnell JP, Nair KS (2008). Diabetes.

[47] Welle S, Brooks AI, Delehanty JM, Needler N, Thornton CA (2003). Physiol Genomics.

[48] Welle S, Brooks AI, Delehanty JM, Needler N, Bhatt K, Shah B, Thornton CA (2004). Exp Gerontol.

[49] Rassoul RA, Alves S, Pantesco V, Vos JD, Michel B, Perret M, Mestre-Francés N, Verdier J-M, Devau G (2010). PLoS ONE.

[50] Pang WW, Price EA, Sahoo D, Beerman I, Maloney WJ, Rossi DJ, Schrier SL, Weissman IL (2011). Proc Natl Acad Sci.

[51] Raue U, Trappe TA, Estrem ST, Qian H-R, Helvering LM, Smith RC, Trappe S (2012). J Appl Physiol.

[52] Liu D, Sartor MA, Nader GA, Pistilli EE, Tanton L, Lilly C, Gutmann L, IglayReger HB, Visich PS, Hoffman EP, Gordon PM (2013). J Gerontol Ser A.

[53] Cao J, Agrawal A, Sharman E, Jia Z, Gupta S (2014). PLoS ONE.

[54] Lu T, Aron L, Zullo J, Pan Y, Kim H, Chen Y, Yang T-H, Kim H-M, Drake D, Liu XS, Bennett DA, Colaiácovo MP, Yankner BA (2014). Nature.

[55] Hou Y, Dan X, Babbar M, Wei Y, Hasselbalch SG, Croteau DL, Bohr VA (2019). Nat Rev Neurol.

[56] Jo M, Lee S, Jeon YM, Kim S, Kwon Y, Kim HJ (2020). Exp Mol Med.

[57] Jeon GS, Shim YM, Lee DY, Kim JS, Kang MJ, Ahn SH, Shin JY, Geum D, Hong YH, Sung JJ (2007). Mol Neurobiol.

[58] Neumann M, Sampathu DM, Kwong LK, Truax AC, Micsenyi MC, Chou TT, Bruce J, Schuck T, Grossman M, Clark CM, McCluskey LF, Miller BL, Masliah E, Mackenzie IR, Feldman H, Feiden W, Kretzschmar HA, Trojanowski JQ, Lee VMY (2006). Science.

[59] Robertson J, Sanelli T, Xiao S, Yang W, Horne P, Hammond R, Pioro EP, Strong MJ (2007). Neurosci Lett.

[60] Noto YI, Shibuya K, Sato Y, Kanai K, Misawa S, Sawai S, Mori M, Uchiyama T, Isose S, Nasu S, Sekiguchi Y, Fujimaki Y, Kasai T, Tokuda T, Nakagawa M, Kuwabara S (2011). Amyotroph Lateral Scler.

[61] Verstraete E, Kuiperij HB, Van Blitterswijk MM, Veldink JH, Schelhaas HJ, Van Den Berg LH, Verbeek MM (2012). Amyotroph Lateral Scler.

[62] Nikopoulou C, Parekh S, Tessarz P (2019). Biol Chem.

[63] O’Laughlin R, Jin M, Li Y, Pillus L, Tsimring LS, Hasty J, Hao N (2020). Transl Med Aging.

[64] Nordstokke DW, Zumbo BD (2007). J Educ Res Policy Stud.

[65] Leek JT, Scharpf RB, Bravo HC, Simcha D, Langmead B, Johnson WE, Geman D, Baggerly K, Irizarry RA (2010). Nat Rev Genet.

[66] Haghverdi L, Lun ATL, Morgan MD, Marioni JC (2018). Nat Biotechnol.

[67] Baldari S, Di Rocco G, Piccoli M, Pozzobon M, Muraca M, Toietta G (2017). Int J Mol Sci.

[68] Di Micco R, Krizhanovsky V, Baker D, d’Adda di Fagagna F (2020). Nat Rev Mol Cell Biol.

[69] Fulop T, Larbi A, Dupuis G, Le Page A, Frost EH, Cohen AA, Witkowski JM, Franceschi C (1960). Front Immunol.

[70] Ferrucci L, Fabbri E (2018). Nat Rev Cardiol.

[71] Thun MJ, Henley SJ, Gansler T (2004). Novartis Found Symp.

[72] Libby P, Ridker PM, Maseri A. Inflammation and atherosclerosis. Circulation. 2002;105:1135. 10.1161/hc0902.104353 11877368

[73] Howlader N, Noone AM, Krapcho M, Miller D, Brest A, Yu M, Ruhl J, Tatalovich Z, Mariotto A, Lewis DR, Chen HS, Feuer EJ. SEER Cancer Statistics Review. 1975-2017. National Cancer Institute. https://seer.cancer.gov/csr/1975_2017/. Accessed Apr 2020.

[74] Su CW, Lin CW, Yang WE, Yang SF. Ther Adv Med Oncol. 2019;11. 10.1177/1758835919864247 PMC6637839 31360238

[75] Hahn SA, Schutte M, Shamsul Hoque ATM, Moskaluk CA, Da Costa LT, Rozenblum E, Weinstein CL, Fischer A, Yeo CJ, Hruban RH, Kern SE (1996). Science.

[76] Müller A, Homey B, Soto H, Ge N, Catron D, Buchanan ME, McClanahan T, Murphy E, Yuan W, Wagner SN, Barrera JL, Mohar A, Verástegui E, Zlotnik A (2001). Nature.

[77] Gupta SK, Lysko PG, Pillarisetti K, Ohlstein E, Stadel JM (1998). J Biol Chem.

[78] Devine SM, Flomenberg N, Vesole DH, Liesveld J, Weisdorf D, Badel K, Calandra G, DiPersio JF (2004). J Clin Oncol.

[79] Broxmeyer HE, Orschell CM, Clapp DW, Hangoc G, Cooper S, Plett PA, Liles WC, Li X, Graham-Evans B, Campbell TB, Calandra G, Bridger G, Dale DC, Srour EF. Rapid mobilization of murine and human hematopoietic stem and progenitor cells with AMD3100, a CXCR4 antagonist. J Exp Med. 2005;201:1307. 10.1084/jem.20041385 PMC2213145 15837815

[80] Sandgren EP, Schroeder JA, Qui TH, Palmiter RD, Brinster RL, Lee DC. Rapid mobilization of murine and human hematopoietic stem and progenitor cells with AMD3100, a CXCR4 antagonist. Cancer Res. 1995;55.

[81] Takagi H, Sharp R, Takayama H, Anver MR, Ward JM, Merlino G. Cancer Res. 1993;53. 8364928

[82] Zhang X, Ge YL, Zhang SP, Yan P, Tian RH. Downregulation of KDR expression induces apoptosis in breast cancer cells. Cell Mol Biol Lett. 2014;19:527. 10.2478/s11658-014-0210-8 PMC6276020 25182240

[83] Takahashi Y, Kitadai Y, Bucana CD, Cleary KR, Ellis LM. Cancer Res. 1995;55. 7664263

[84] Safina A, Vandette E, Bakin AV (2007). Oncogene.

[85] Shen L, Qu X, Ma Y, Zheng J, Chu D, Liu B, Li X, Wang M, Xu C, Liu N, Yao L, Zhang J. Tumor suppressor NDRG2 tips the balance of oncogenic TGF-β via EMT inhibition in colorectal cancer. Oncogenesis. 2014;3:e86. 10.1038/oncsis.2013.48 PMC3940918 24492480

[86] Sandgren EP, Luetteke NC, Qiu TH, Palmiter RD, Brinster RL, Lee DC. Transforming growth factor alpha dramatically enhances oncogene-induced carcinogenesis in transgenic mouse pancreas and liver. Mol Cell Biol. 1993. 10.1128/mcb.13.1.320-330.1993 PMC358911 8417334

[87] Vaure C, Liu Y. Front Immunol. 2014, 5. 10.3389/fimmu.2014.00316 PMC4090903 25071777

[88] Yu L, Wang L, Chen S (2010). J Cell Mol Med.

[89] Molinaro A, Holst O, Di Lorenzo F, Callaghan M, Nurisso A, D’Errico G, Zamyatina A, Peri F, Berisio R, Jerala R, Jiménez-Barbero J, Silipo A, Martín-Santamaría S (2015). Chem-Eur J.

[90] Letiembre M, Hao W, Liu Y, Walter S, Mihaljevic I, Rivest S, Hartmann T, Fassbender K (2007). Neuroscience.

[91] Seo S-W, Park S-K, Oh S-J, Shin OS (2018). PLoS ONE.

[92] Calvo-Rodríguez M, de la Fuente C, García-Durillo M, García-Rodríguez C, Villalobos C, Núñez L (2017). J Neuroinflammation.

[93] Takizawa H, Fritsch K, Kovtonyuk LV, Saito Y, Yakkala C, Jacobs K, Ahuja AK, Lopes M, Hausmann A, Hardt W-D, Gomariz Á, Nombela-Arrieta C, Manz MG (2017). Cell Stem Cell.

[94] Xu M, Tchkonia T, Ding H, Ogrodnik M, Lubbers ER, Pirtskhalava T, White TA, Johnson KO, Stout MB, Mezera V, Giorgadze N, Jensen MD, LeBrasseur NK, Kirkland JL (2015). Proc Natl Acad Sci U S A.

[95] Banerjee S, Biehl A, Gadina M, Hasni S, Schwartz DM (2017). Drugs.

[96] Tilstra JS, Clauson CL, Niedernhofer LJ, Robbins PD (2011). Aging Dis.

[97] Haigis MC, Yankner BA (2010). Mol Cell.

[98] Chung HY, Kim DH, Lee EK, Chung KW, Chung S, Lee B, Seo AY, Chung JH, Jung YS, Im E, Lee J, Kim ND, Choi YJ, Im DS, Yu BP (2019). Aging Dis.

[99] Liu T, Zhang L, Joo D, Sun SC (2017). Signal Transduct Target Ther.

[100] Tominaga K, Suzuki HI (2019). Int J Mol Sci.

[101] Biere AL, Ostaszewski B, Stimson ER, Hyman BT, Maggio JE, Selkoe DJ (1996). J Biol Chem.

[102] Boada M, López O, Núñez L, Szczepiorkowski ZM, Torres M, Grifols C, Páez A (2019). Alzheimers Dement Transl Res Clin Interv.

[103] Lemere CA, Masliah E (2010). Nat Rev Neurol.

[104] Patidar GK, Land KJ, Vrielink H, Rahimi-Levene N, Dann EJ, Al-Humaidan H, Spitalnik SL, Dhiman Y, So-Osman C, Hindawi SI (2021). Vox Sang.

[105] Wang P, Wander CM, Yuan CX, Bereman MS, Cohen TJ (2017). Nat Commun.

[106] Pandya VA, Patani R (2020). Brain.

[107] Al Dubayee MS, Alayed H, Almansour R, Alqaoud N, Alnamlah R, Obeid D, Alshahrani A, Zahra MM, Nasr A, Al-Bawab A, Aljada A (2018). Front Endocrinol.

[108] Jiang K, Frank MB, Chen Y, Osban J, Jarvis JN (2013). Arthritis Res Ther.

[109] Sotillo E, Barrett DM, Black KL, Bagashev A, Oldridge D, Wu G, Sussman R, Lanauze C, Ruella M, Gazzara MR, Martinez NM, Harrington CT, Chung EY, Perazzelli J, Hofmann TJ, Maude SL, Raman P, Barrera A, Gill S, Lacey SF, Melenhorst JJ, Allman D, Jacoby E, Fry T, Mackall C, Barash Y, Lynch KW, Maris JM, Grupp SA, Thomas-Tikhonenko A (2015). Cancer Discov.

[110] Braitch M, Harikrishnan S, Robins RA, Nichols C, Fahey AJ, Showe L, Constantinescu CS (2009). Acta Neurol Scand.

[111] Maher IE, Griffith JE, Lau Q, Reeves T, Higgins DP (2014). PeerJ.

[112] Lowe DB, Taylor JL, Storkus WJ (2014). Methods Mol Biol.

[113] Maingat FG, Polyak MJ, Paul AM, Vivithanaporn P, Noorbakhsh F, Ahboucha S, Baker GB, Pearson K, Power C (2013). FASEB J.

[114] Petre CE, Sin S-H, Dittmer DP (1912). J Virol.

[115] Ocampo A, Reddy P, Martinez-Redondo P, Platero-Luengo A, Hatanaka F, Hishida T, Li M, Lam D, Kurita M, Beyret E, Araoka T, Vazquez-Ferrer E, Donoso D, Roman JL, Xu J, Rodriguez Esteban C, Nuñez G, Nuñez Delicado E, Campistol JM, Guillen I, Guillen P, Izpisua Belmonte JC (2016). Cell.

[116] Metsalu T, Vilo J (2015). Nucleic Acids Res.

[117] Lim T-S, Loh W-Y (1996). Comput Stat Data Anal.

