# Correction to: Old plasma dilution reduces human biological age: a clinical study

**DOI:** 10.1007/s11357-023-00997-x

**Published:** 2023-11-06

**Authors:** Daehwan Kim, Dobri D. Kiprov, Connor Luellen, Michael Lieb, Chao Liu, Etsuko Watanabe, Xiaoyue Mei, Kaitlin Cassaleto, Joel Kramer, Michael J. Conboy, Irina M. Conboy

**Affiliations:** 1 Department of Bioengineering and QB3 Institute, University of California, Berkeley, CA 94720 USA; 2 Global Apheresis, Mill Valley, CA USA; 3 Biophysics, University of California, Berkeley, Berkeley, CA 94720 USA; 4 Brain Aging Center, UCSF, San Francisco, USA

**Correction to: GeroScience (2022) 44:2701–2720**

**https://doi.org/10.1007/s11357-022-00645-w**

The original version of this article unfortunately contained a typo in the ESM file.

There is a typo in the Supplementary file 2 Excel: the same serum sample, Older_Control Male 76, is listed twice; accordingly, there were 15 (not 16) old serum samples in the study.

A corrected Supplementary file 2 added in this paper.

### Supplementary Information

Below is the link to the electronic supplementary material.

Supplementary file1 (XLSX 10.2 KB)

