# Supplementary material for: Old plasma dilution reduces human biological age: a clinical study
Source: GeroScience. 2022 Aug 24;44(6):2701–20. doi: 10.1007/s11357-022-00645-w (PMC9398900; doi:10.1007/s11357-022-00645-w)
Supplement: Supplementary file 1 — Supplementary file1 (DOCX 2094 KB) [file 11357_2022_645_MOESM1_ESM.docx]

**Supplementary data**


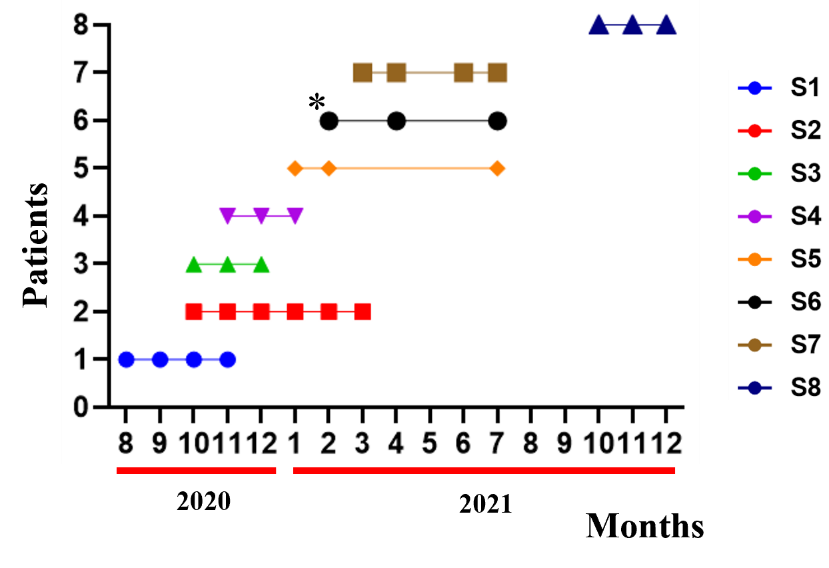


**Supplementary Figure 1. The graph shows the dates and intervals at which the samples were collected.**

* 2 TPE procedures were done in one week; the comparative analyses were on the rounds of TPE procedure that were separated from each other by 1 month or more. Of note, R0 and R1 blood samples of Subject 1 were of poor technical quality, so we started with the R2 sample, which is the first dot on the graph.


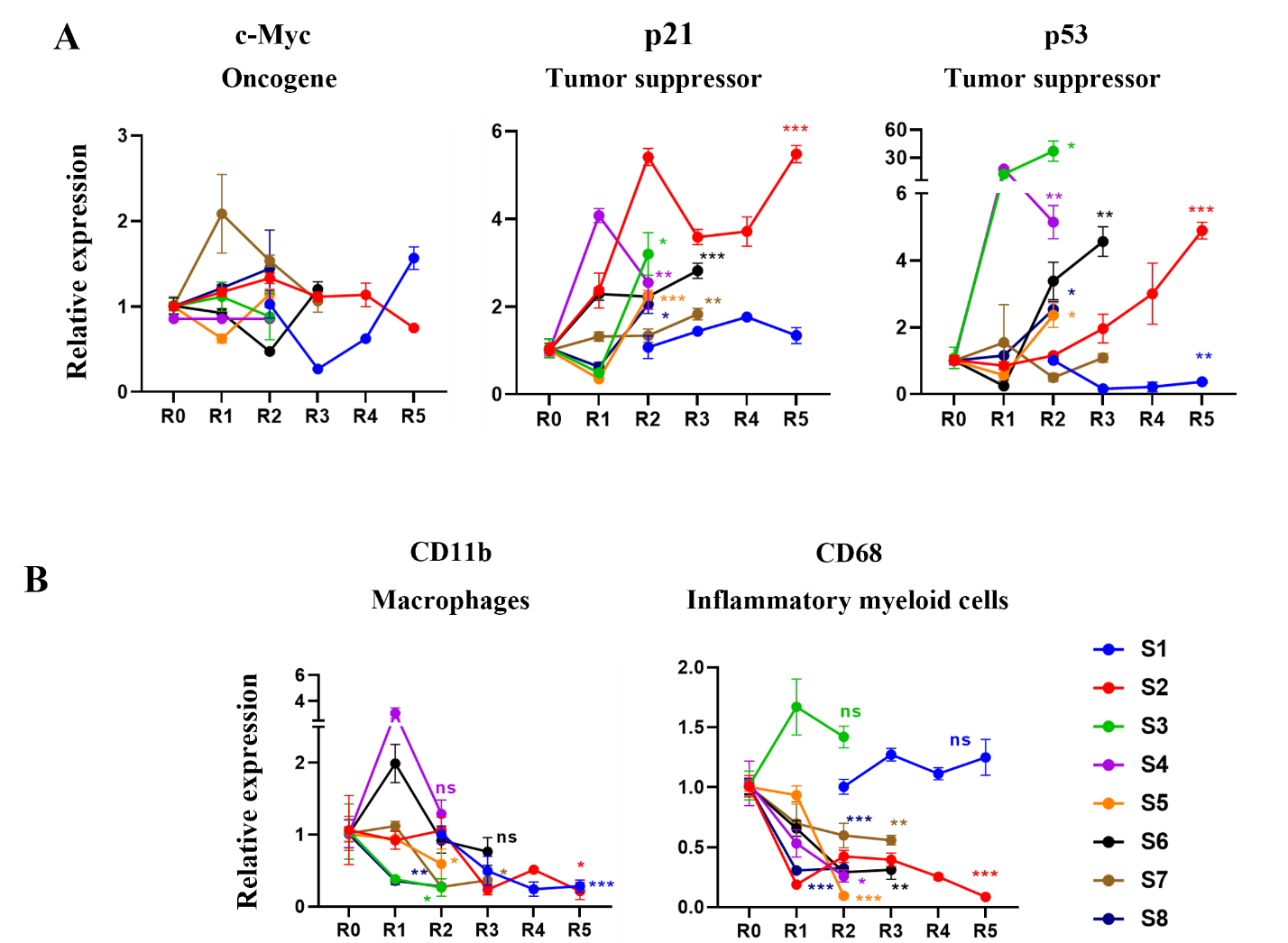


**Supplementary Figure 2. The expression profiling of tumor related genes and myeloid genes.** A. Although there was no difference in the expression of c-Myc, the expression of p21 and p53 was elevated by rounds of TPE treatment. B. The markers of myeloid genes (CD11b and CD68) become reduced by the rounds of TPE in the old PBMCs. Each gene profiling is performed by qPCR in 3 replicates. *P<0.05, **P<0.01, ***P<0.001, ns = not significant.


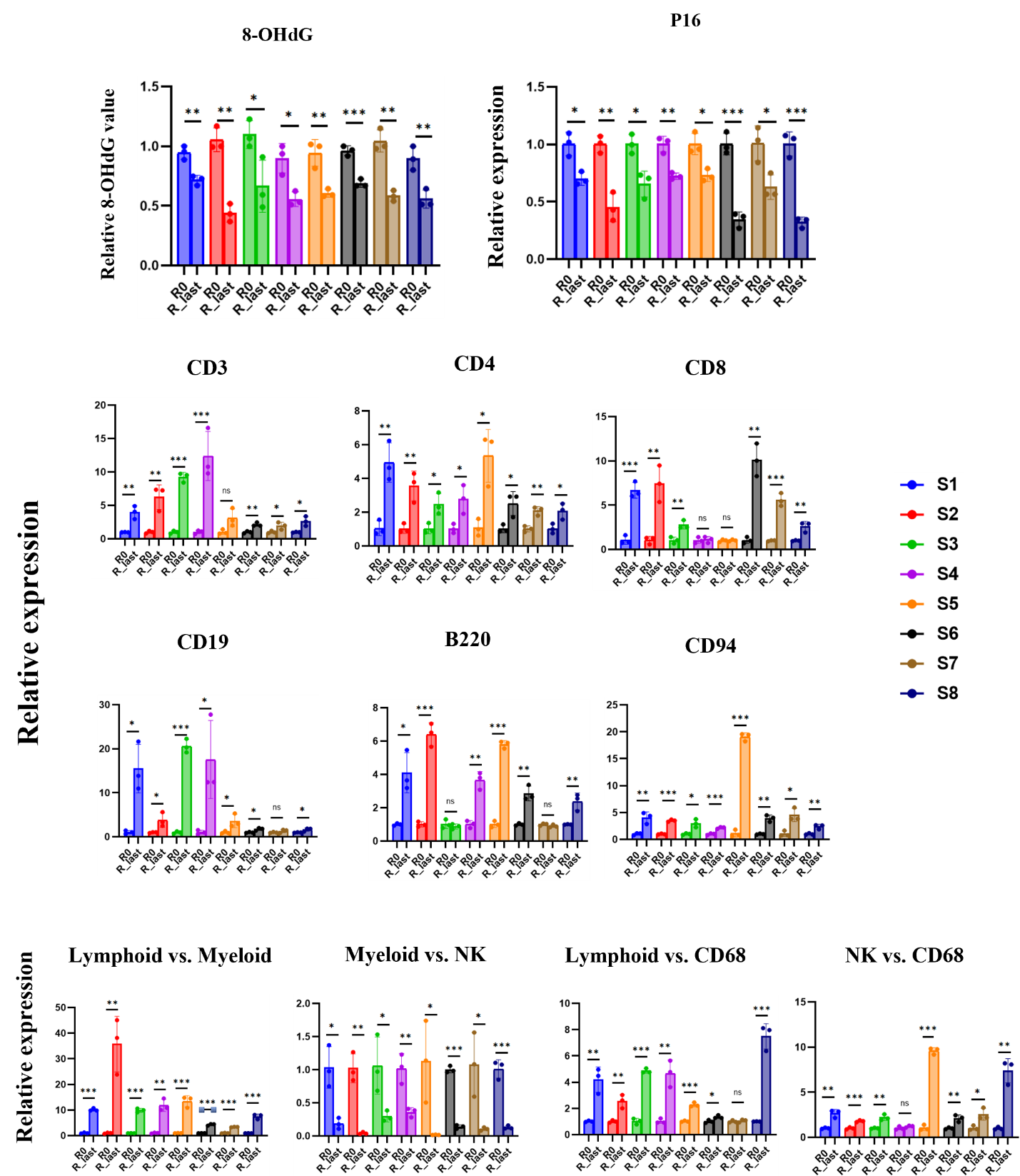


**Supplementary Figure 3. Comparison between R0 and R_last confirm the TPE effect.** The relative values of R0 and R_last in each marker (see Figure 1B-E) were compared and show the TPE effect. Each gene profiling is performed by qRT-PCR or ELISA in 3 replicates for each sample. *P<0.05, **P<0.01, ***P<0.001, ns = not significant.


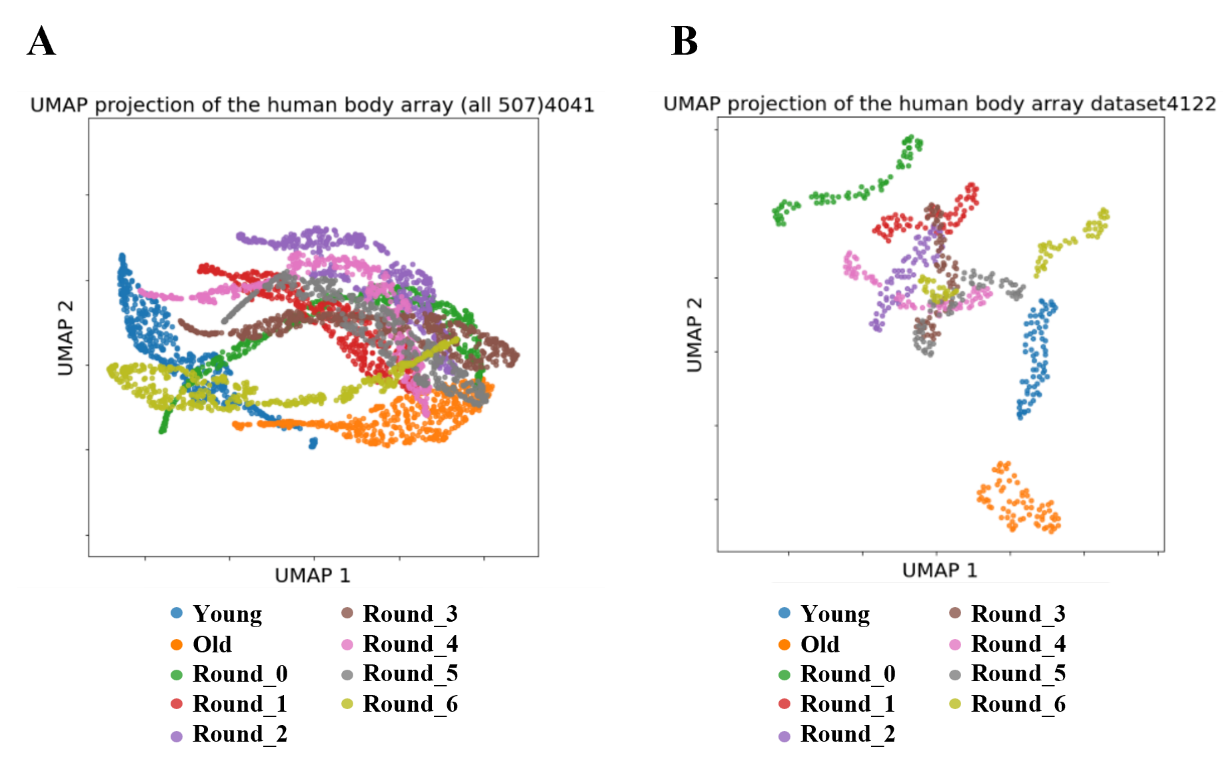


**Supplementary Figure 4. Distinct clustering shifts by rounds of repetitive TPE.** A. The uMAP on whole 507 proteome levels shows complicated interactions among cohorts. B. The uMAP on selected 72 proteins provides comprehensible differences between young and old cohorts. Moreover, 72 proteins are approaching to the young cohort, rather than the old cohort by rounds of repetitive TPE.


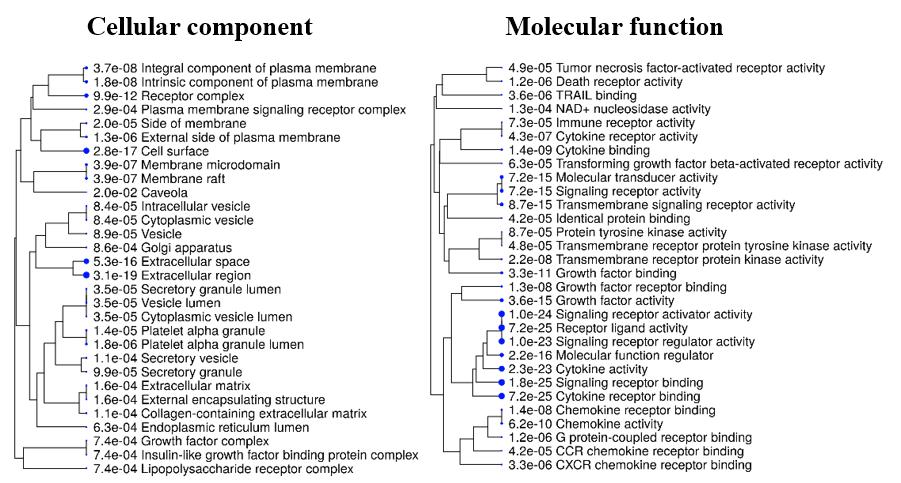


**Supplementary Figure 5. Top 30 hierarchical clustering tree of cellular component and molecular function.**


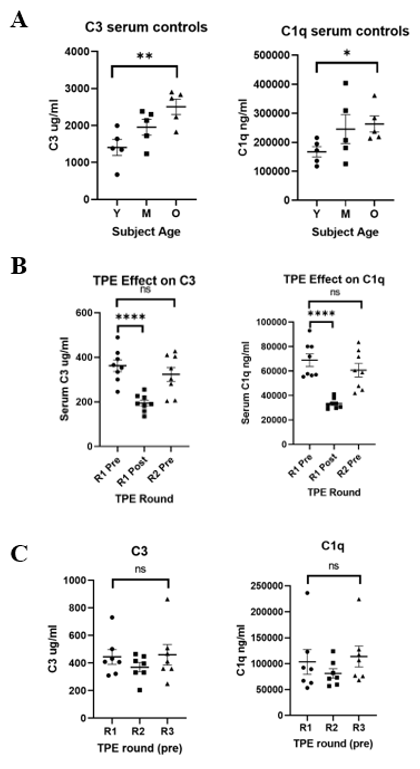


**Supplementary Figure 6. The effects of TPE on C3 and C1q complement levels.** A. C3 and C1q are assayed in three different aged control samples. There is a significant increase in both C3 and C1q protein levels in old controls as compared to young controls. B. Levels of C3 and C1q are analyzed via ELSA in serum samples that are taken immediately before (pre) or after (post) a TPE procedure. TPE significantly reduces both C1q and C3 levels in the post group compared to pre group. However, there is no significant change to C3 and C1q levels by the next round of TPE across all individuals treated. C. C3 and C1q are assayed in serum samples collected from three different TPE rounds. C3 and C1q protein levels show no significant or long-lasting change among rounds of TPE treatment. Each data profiling is performed by ELISA in 3 replicates. **P*<0.05, ***P*<0.01 ****P*<0.001. ns = not significant.


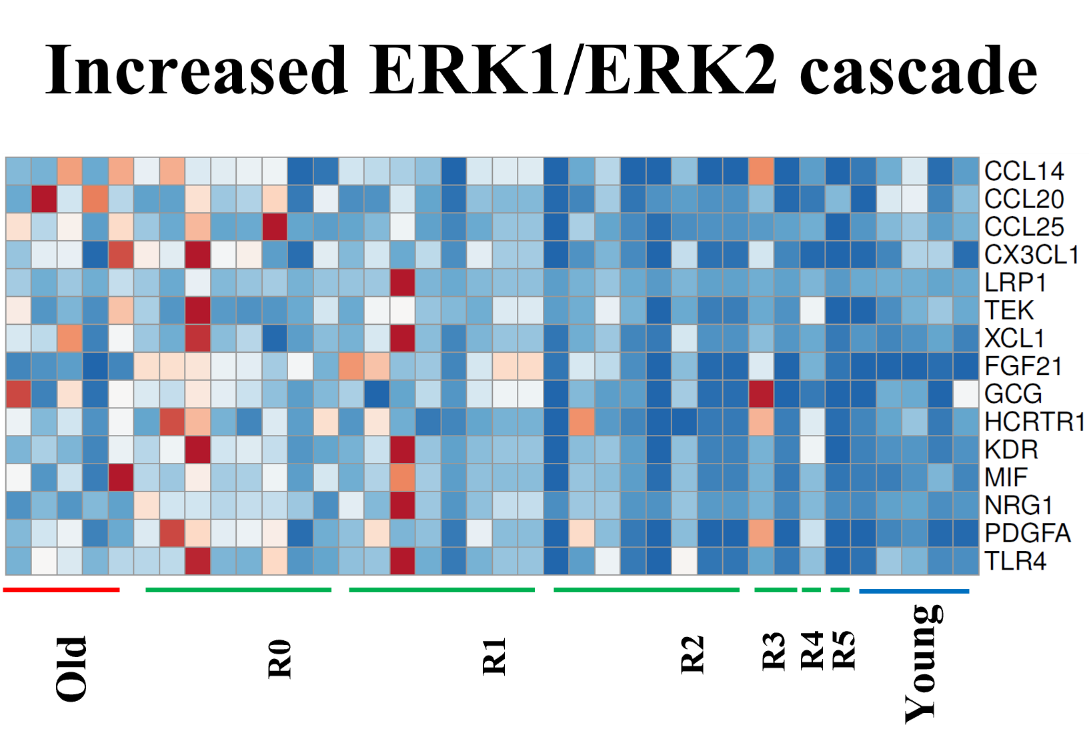


**Supplementary Figure 7. The heatmap of Erk1/Erk2 cascade (*p*= 5.50E-14).** The ERK cascade proteins, that when increased promote aging or senescence, CCL20, CCL25, CX3CL1, and TLR4, were all normalized to their younger levels by the rounds of TPE.

**
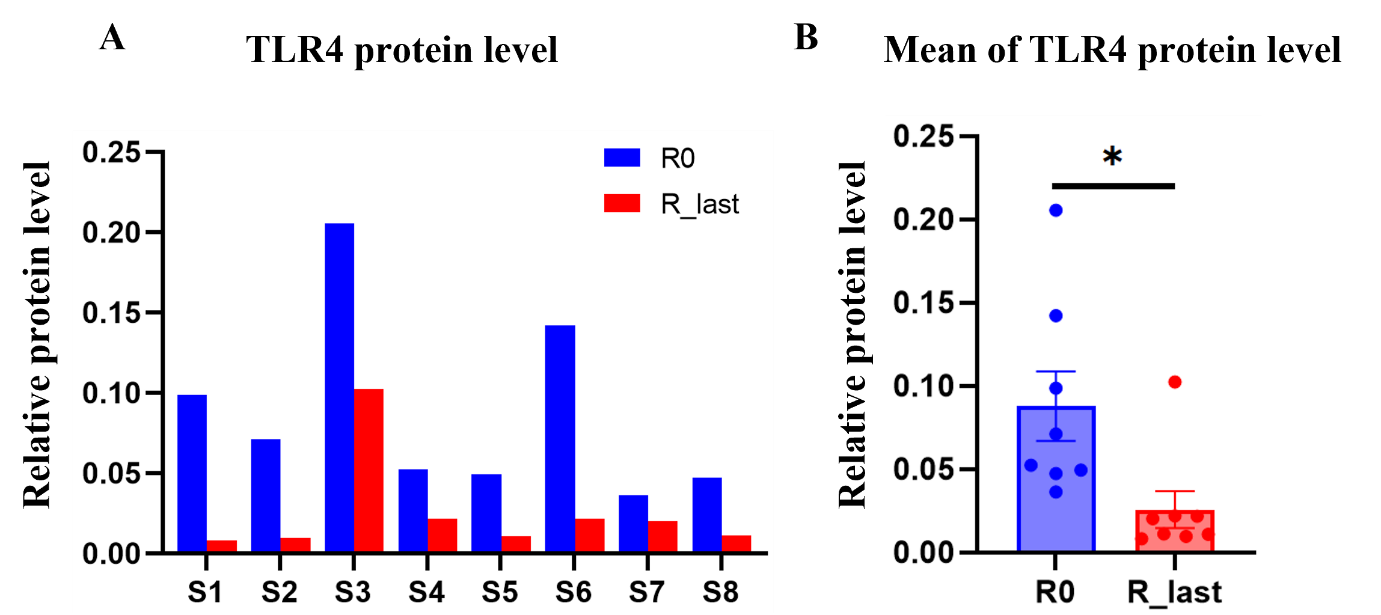
**

**Supplementary Figure 8. The protein levels of TRL4 in the TPE groups.** A. The TLR4 protein levels of 8 individuals before (R0) and after (R-last) TPE. B. The levels for each subject and the Mean for R0 and R-last are shown. N^R0^=8, N^R_last^ = 8, *P<0.05.

**
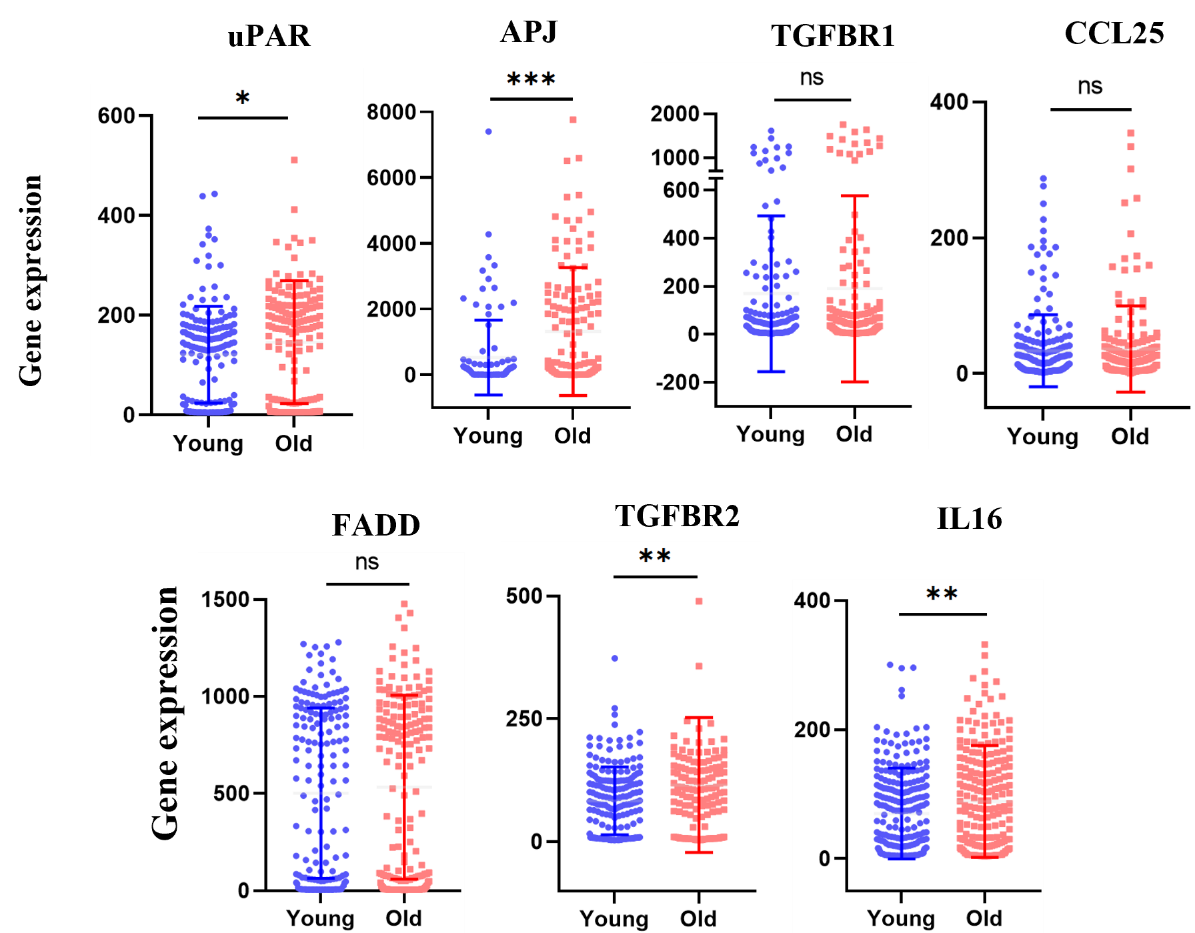
**

**Supplementary Figure 9. Gene expressions of representative genes with SD.** The expressions of uPAR, APJ, TGFBR2 and IL16 are increased significantly in the Old groups but there are no differences in TGFBR1, FADD, and CCL25 expressions between the young and the old groups, *P<0.05, **P<0.01, ***P<0.001, ns = not significant, N=349. Interestingly, the SD (bars) suggest that the biological noise of the representative genes increases with age.

**
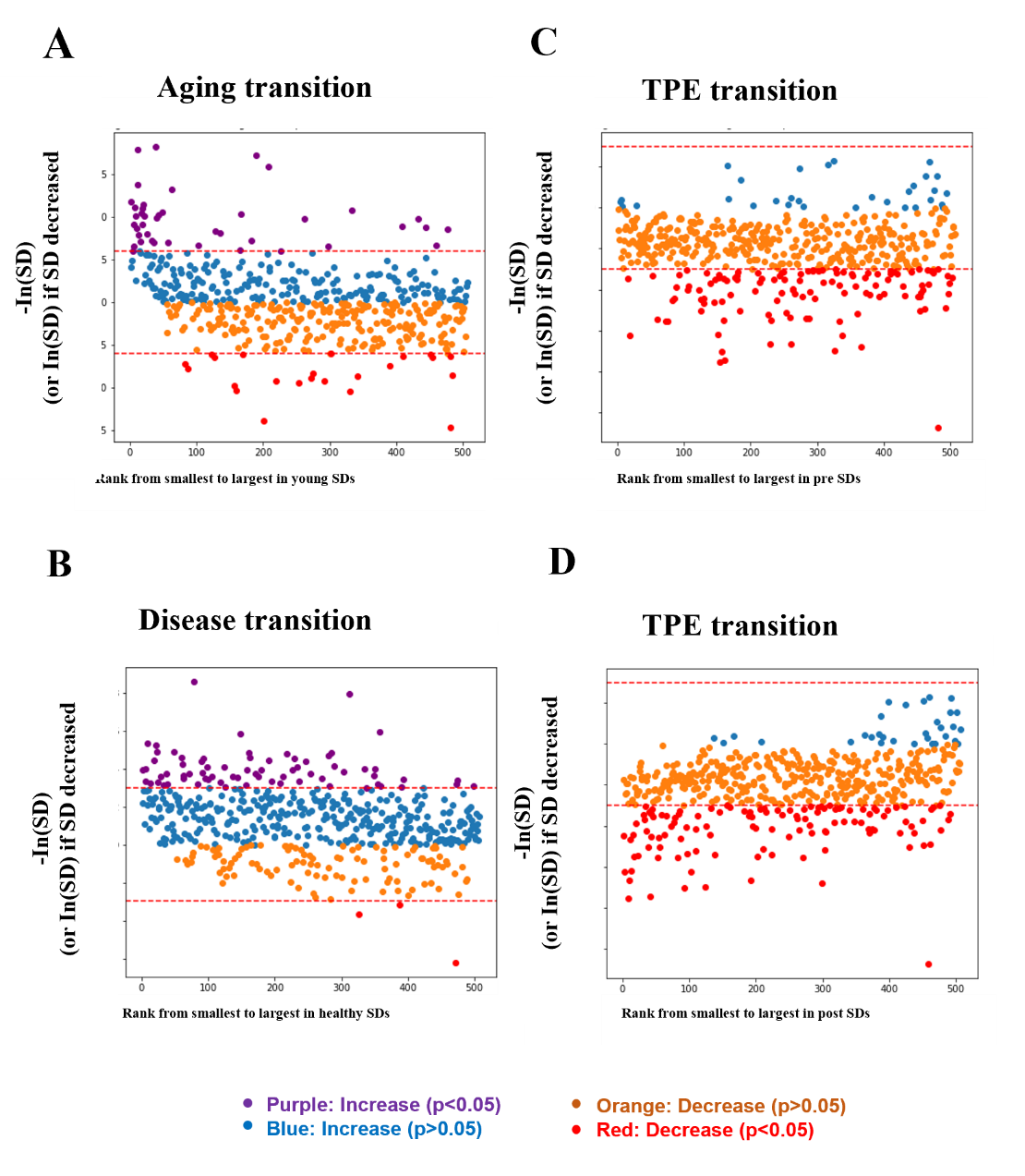
**

**Supplementary Figure 10.** V**isualization of the p values found with various forms of the Levene test.** A and B. Visualization of the p values found with the Levene test when comparing variances of proteins between the compared groups. Increases in variance between the healthy group and disease group are shown in blue, or purple if they are significant, and are plotted as -log(p value). Decreases in variance between the healthy group and disease group are shown in orange, or red if they are significant, and are plotted as log(p value). ± log (0.05) is displayed as a red dashed line. The x axis is the rank of the SD in the young group (A) or the healthy group (B). C and D. A visualization of the p values found with various forms of the Levene test when comparing variances of proteins between people before and after TPE treatment. Increases in variance between the pre- group and post treatment group are shown in blue, or purple if they are significant, and are plotted as -log(p value). Decreases in variance between the pre- group and post treatment group are shown in orange, or red if they are significant, and are plotted as log(p value). ± log (0.05) is displayed as a red dashed line. The x axis is the rank of the SD in the pre- group (C) or the post treatment group (D).

**
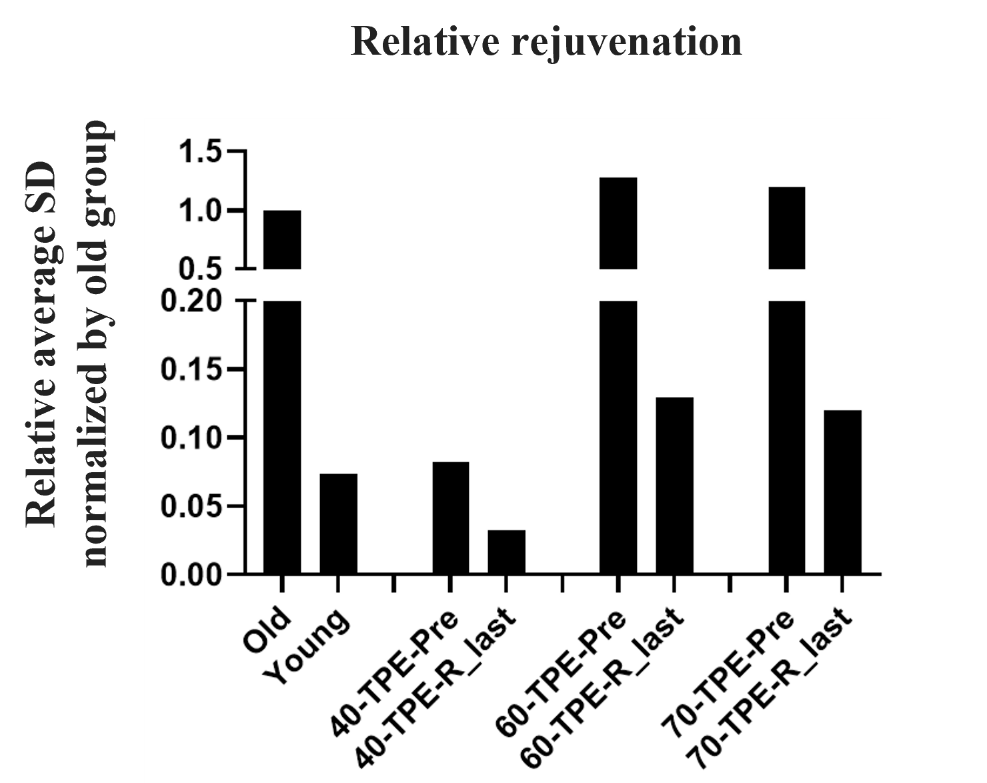
**

**Supplementary Figure 11. The relative rejuvenation.** The average of the selected proteins in each group is normalized by the old group. The net change between groups shows relative rejuvenation. The age (40, 60, 70) indicates the lowest age decade of each age group.

**Supplementary Table 1:** Excel File shows the ages and genders of all subjects of these studies.
